# Gene-based association tests using GWAS summary statistics and incorporating eQTL

**DOI:** 10.1038/s41598-022-07465-0

**Published:** 2022-03-03

**Authors:** Xuewei Cao, Xuexia Wang, Shuanglin Zhang, Qiuying Sha

**Affiliations:** 1 Department of Mathematical Sciences, Michigan Technological University, Houghton, MI 49931 USA; 2 Department of Mathematics, University of North Texas, Denton, TX USA

**Keywords:** Computational biology and bioinformatics, Genetics

## Abstract

Although genome-wide association studies (GWAS) have been successfully applied to a variety of complex diseases and identified many genetic variants underlying complex diseases via single marker tests, there is still a considerable heritability of complex diseases that could not be explained by GWAS. One alternative approach to overcome the missing heritability caused by genetic heterogeneity is gene-based analysis, which considers the aggregate effects of multiple genetic variants in a single test. Another alternative approach is transcriptome-wide association study (TWAS). TWAS aggregates genomic information into functionally relevant units that map to genes and their expression. TWAS is not only powerful, but can also increase the interpretability in biological mechanisms of identified trait associated genes. In this study, we propose a powerful and computationally efficient gene-based association test, called Overall. Using extended Simes procedure, Overall aggregates information from three types of traditional gene-based association tests and also incorporates expression quantitative trait locus (eQTL) information into a gene-based association test using GWAS summary statistics. We show that after a small number of replications to estimate the correlation among the integrated gene-based tests, the *p* values of Overall can be calculated analytically. Simulation studies show that Overall can control type I error rates very well and has higher power than the tests that we compared with. We also apply Overall to two schizophrenia GWAS summary datasets and two lipids GWAS summary datasets. The results show that this newly developed method can identify more significant genes than other methods we compared with.

## Introduction

Although genome-wide association studies (GWAS) have successfully identified thousands of single nucleotide polymorphisms (SNPs) associated with a wide range of complex human traits^1,2^, there is a common limitation in which GWAS focus on only a single genetic variant with a trait at a time. This limitation may limit the power to identify clinically or biologically significant genetic associations^3^. Furthermore, many genome-wide significant genetic variants are in linkage disequilibrium (LD). Different LD patterns can cause non-replicated results of the same variant in different populations^4,5^. Therefore, several powerful gene-based statistical association tests, in which the genetic information of all genetic variants in a gene is combined to obtain more informative results, have been developed, such as the Burden Test (BT)^6^, the Sequence Kernel Association Test (SKAT)^7^, and the Optimized SKAT (SKATO)^8^.

When individual-level genotype and phenotype data are not available, the traditional gene-based association tests, BT, SKAT, and SKATO, can be extended by using GWAS summary statistics^9^. Currently, there are many GWAS summary statistics available in public resources^10^. In GWAS summary statistics, the Z-scores of genetic variants in a gene are assumed to asymptotically follow a multivariate normal distribution with a correlation matrix among all genetic variants in a gene under the null hypothesis^11^, where the correlation matrix can be estimated by LD among the genetic variants in the gene^12,13^. When individual-level data are not available, LD is usually estimated using external reference panels^14,15^ (i.e., 1000 Genomes Project^16^). Due to the small sample size of reference panels used to estimate LD, statistical noise (i.e., inflated type I error rates or large numbers of false positives) often exists which needs to be accounted for^17,18^. One way to reduce the statistical noise is to correct the estimated LD by a regularization procedure^19^. In the regularization procedure, a statistical white Gaussian noise is added to the LD matrix which is estimated by a reference panel. After correcting the estimated LD by the regularization procedure, we can assume that, under the null hypothesis, the Z-scores from GWAS summary statistics asymptotically follow a multivariate normal distribution with the correlation matrix being the corrected LD matrix among the genetic variants in a gene.

To increase statistical power in identifying genes that are associated with complex diseases, PrediXcan^20^ and transcriptome-wide association study^12,21^ (TWAS) were developed by incorporating expression quantitative trait locus (eQTL) data into GWAS. As pointed out by Zhang et al.^15^, PrediXcan and TWAS can be viewed as a simple weighted linear combination of genetic variants with an eQTL—derived weight. In fact, the genetic architecture of complex traits is rarely known in advance and is likely to vary from one region to another across the genome and from one trait to another^15^. Therefore, only considering one single eQTL—derived weight, such as in PrediXcan and TWAS, may lose statistical power in identifying significant genes. Zhang et al.^15^ developed an omnibus test (OT) using Cauchy combination method to integrate association evidence obtained by BT, SKAT, and SKATO based on GWAS summary data with multiple eQTL‐derived weights. They showed that OT using multiple eQTL—derived weights had some potential advantages.

Inspired by the advantage of OT, in this paper, we propose a more powerful and computationally efficient method, called Overall, to aggregate the information from three types of traditional gene-based association tests (BT, SKAT, SKATO) with multiple eQTL—derived weights using GWAS summary statistics. To combine information from the three gene-based association tests, the Overall method utilizes the extended Simes procedure^5,22^. To apply the Overall method, we first need to estimate the correlation matrix among the three gene-based association tests with eQTL—derived weights under the null hypothesis. We provide an estimation method using a replication procedure^23,24^. The replication procedure only needs to be performed once to obtain the correlation matrix for each gene. Then, the p-values of Overall can be analytically computed without using permutations. To calculate the p-values of the three types of gene-based association tests (BT, SKAT, SKATO) using GWAS summary statistics with eQTL—derived weights, we use the “sumFREGAT” package in R (https://cran.r-project.org/web/packages/sumFREGAT/index.html)^9^. Once we obtain the p-values of these three tests, the p-value of our proposed method can be easily calculated using its theoretical distribution. Extensive simulation studies show that Overall can control type I error rates well and has higher power than the comparison methods across most of the simulation settings. Similar to Zhang et al.^15^, we apply our method to two schizophrenia (SCZ) and two lipids trait (HDL) GWAS summary data sets. Compared with OT and other tests, the proposed method can identify more significant genes. More interestingly, some significant genes reported by GWAS catalog are only identified by our proposed method.

## Statistical models and methods

### Statistical models

Consider a set of $M$ genetic variants in a gene. Let ${\user2{Z}} = \left( {Z_{1} , \ldots ,Z_{M} } \right)^{T}$ be an $M \times 1$ vector of Z-scores of the $M$ genetic variants. Note that the Z-scores is either directly provided by publicly available GWAS summary statistics or calculated from a GWAS individual-level genotype and phenotype data set. We are interested in testing the null hypothesis $H_{0}$ that none of the genetic variants in the gene is associated with a trait, whereas the alternative hypothesis is that at least one genetic variant in the gene is associated with a trait. Following Svishcheva et al.^9^, Gusev et al.^12^, and Yang et al.^25^, we assume $\user2{Z} = \left( {Z_{1} , \ldots ,Z_{M} } \right)^{T} \sim {\text{MVN}}\left( {{\varvec{0}},{\mathbf{R}}} \right)$ under the null hypothesis, where ${\mathbf{R}}$ is the correlation matrix among ${\varvec{Z}}$, which can be estimated by LD among the genetic variants in the gene^12,13^. If individual-level data are not available, LD can be estimated using external reference panels (i.e., 1000 Genomes Project^16^). However, if the sample size of a reference panel is small, LD may not be estimated correctly so that it will induce statistical noise (i.e., inflated type I error rates or large numbers of false positives)^17,18^. One way to correct the estimated LD is to use a regularization procedure by adding a statistical white Gaussian noise^9,19^. Let ${\mathbf{I}}_{M}$ be an $M \times M$ identity matrix, and the corrected correlation matrix ${\mathbf{U}}$ can be defined as

$${\mathbf{U}} = a{\mathbf{R}} + \left( {1 - a} \right){\mathbf{I}}_{M} ,\quad 0 \le a \le 1,$$

where $a$ is a scalar tuning parameter which represents the coefficient of proportionality between the corrected correlation matrix ${\mathbf{U}}$ and the original ${\mathbf{R}}$ estimated using an external reference panel. The optimal tuning parameter $a$ can be estimated by maximizing the log-likelihood function of the distribution of ${\varvec{Z}} \sim {\text{MVN}}\left( {{\varvec{0}},{\mathbf{U}}} \right)$, that is,

$$\hat{a} = \mathop {\arg \max }\limits_{{a \in \left[ {0,1} \right]}} \left\{ {\log \left( {L\left( {{\varvec{Z}}:{\varvec{0}},{\mathbf{U}}} \right)} \right)} \right\}.$$

Then the corrected correlation matrix ${\hat{\mathbf{U}}} = \hat{a}{\mathbf{R}} + \left( {1 - \hat{a}} \right){\mathbf{I}}_{M}$. Therefore, under the null hypothesis, we consider ${\varvec{Z}} = \left( {Z_{1} , \ldots ,Z_{M} } \right)^{T} \sim {\text{MVN}}\left( {{\varvec{0}},{\hat{\mathbf{U}}}} \right)$.

Suppose that there are a total of $K$ different eQTL—derived weights from gene expression data (i.e., Genotype-Tissue Expression (GTEx) project (https://gtexportal.org/home/)), denoted as ${\hat{\mathbf{W}}}_{k} = {\text{diag}} \left( {\hat{W}_{1}^{k} , \ldots ,\hat{W}_{M}^{k} } \right)$ for $k = 0,1, \ldots ,K$, where ${\hat{\mathbf{W}}}_{0} = {\text{diag}} \left( {1, \ldots ,1} \right)$ represents a status without using any weights. In order to avoid the influence of the scale among genetic variants within each weight, we first standardize the eQTL—derived weights ${\mathbf{W}}_{k}$ as $W_{m}^{k} = {{\hat{W}_{m}^{k} } \mathord{\left/ {\vphantom {{\hat{W}_{m}^{k} } {\sum\nolimits_{m = 1}^{M} {\left| {\hat{W}_{m}^{k} } \right|} }}} \right. \kern-\nulldelimiterspace} {\sum\nolimits_{m = 1}^{M} {\left| {\hat{W}_{m}^{k} } \right|} }}$ for $m = 1, \ldots ,M$. Based on the $k$th standardized weight ${\mathbf{W}}_{k}$, the weighted Z-score ${\mathbf{W}}_{k} {\varvec{Z}}$ follows a multivariate normal distribution. That is,

$${\mathbf{W}}_{k} {\varvec{Z}} \sim {\text{MVN}} \left( {{\mathbf{0}},{\hat{\mathbf{\Sigma }}}_{k} } \right)\,{\text{and}}\,{\hat{\mathbf{\Sigma }}}_{k} = {\mathbf{W}}_{k} {\hat{\mathbf{U}}\mathbf{W}}_{k} .$$

We extend the three types of gene-based association tests, BT^6^, SKAT^7^, and SKATO^8^, to incorporate the eQTL—derived weights based on GWAS summary statistics^9,26^. For the *k*th eQTL—derived weight, the three gene-based test statistics can be written as

$$\begin{aligned} & Q_{BT}^{k} = \left( {{\varvec{Z}}^{T} {\mathbf{W}}_{k} {\varvec{1}}_{M} } \right)^{2} , \\ & Q_{SKAT}^{k} = \left( {{\mathbf{W}}_{k} {\varvec{Z}}} \right)^{T} {\mathbf{W}}_{k} {\varvec{Z}}, \\ & Q_{SKATO}^{k} = \mathop {\min }\limits_{{\rho \in \left[ {0,1} \right]}} \left\{ {\left( {1 - \rho } \right)Q_{SKAT}^{k} + \rho Q_{BT}^{k} } \right\}, \\ \end{aligned}$$

where ${\varvec{1}}_{M}$ is an $M \times 1$ vector with elements of all 1s. Under the null hypothesis, $Q_{BT}^{k}$ follows a $\chi^{2}$ distribution with 1 degree of freedom; $Q_{SKAT}^{k}$ follows a weighted sum of $\chi^{2}$ distributions with 1 degree of freedom; and $Q_{SKATO}^{k}$ follows a mixture of $\chi^{2}$ distribution^8^. The p-values of these three test statistics can be easily calculated using the “sumFREGAT” package in R (https://cran.r-project.org/web/packages/sumFREGAT/index.html)^9^.

### Overall method

To aggregate information from these three gene-based association tests with multiple eQTL—derived weights, we develop a novel method, called Overall, which utilizes the extended Simes procedure^5,22^. Let $p_{BT}^{k} ,p_{SKAT}^{k} ,p_{SKATO}^{k}$ be the p-values of BT, SKAT, SKATO with *k*th eQTL—derived weight, $k = 0,1, \ldots ,K$, respectively, where $k = 0$ denotes a status without using any weights. Thus, there are a total of $L = 3\left( {K + 1} \right)$ p-values from these three gene-based association tests with different weights. Let $\left( {p_{\left( 1 \right)} , \ldots ,p_{\left( L \right)} } \right)$ be a sequence of the ascending p-values, where $p_{\left( 1 \right)} = \min_{k = 0, \ldots ,K} \left\{ {p_{BT}^{k} ,p_{SKAT}^{k} ,p_{SKATO}^{k} } \right\}$ and $p_{\left( L \right)} = \max_{k = 0, \ldots ,K} \left\{ {p_{BT}^{k} ,p_{SKAT}^{k} ,p_{SKATO}^{k} } \right\}$. Overall combines these $L$ p-values using the extended Simes procedure^5,22^, and the p-value of Overall is defined as

$$p_{overall} = \mathop {Min}\limits_{l = 1, \ldots ,L} \left\{ {\frac{{m_{e} p_{\left( l \right)} }}{{m_{e\left( l \right)} }}} \right\},$$

where $m_{e}$ is the effective number of p-values among the $L$ gene-based association tests with multiple weights, $p_{\left( l \right)}$ is the $l$th element of the ascending p-values, and $m_{e\left( l \right)}$ is the effective number of p-values among the top $l$ association tests. We use a more robust measure to obtain the effective numbers $m_{e}$ and $m_{e\left( l \right)}$, which was proposed by Li et al.^5^. The values of $m_{e\left( l \right)}$ and $m_{e}$ can be estimated as

$$m_{e\left( l \right)} = l - \sum\limits_{i = 1}^{l} {\left[ {\left( {\lambda_{i} - 1} \right)I\left( {\lambda_{i} > 1} \right)} \right]} \,{\text{and}}\,m_{e} = m_{e\left( L \right)} ,$$

where $\lambda_{i}$ denotes the *i*th eigenvalue of the correlation matrix ${{\varvec{\Omega}}}$ of p-values from $L$ association tests with multiple weights (the estimation of ${{\varvec{\Omega}}}$ will be discussed in the next section), $I( \cdot )$ is an indicator function. If the $L$ association tests are independent, all eigenvalues $\lambda_{i}$ equal 1, and $m_{e\left( l \right)} = l$ for $l = 1, \ldots ,L$; if the $L$ association tests are perfectly dependent, then $\lambda_{1} = l$ which is the number of tests used to calculate $m_{e\left( l \right)}$ and the other eigenvalues equal 0. In this case, $m_{e\left( l \right)} = l - \left( {l - 1} \right) = 1$ for $l = 1, \ldots ,L$.

The R codes and a sample data set for the implementation of Overall are available at github https://github.com/xueweic/Overall.

### Estimation of ${{\varvec{\Omega}}}$ under the null hypothesis

To apply our proposed method, we need to estimate the correlation matrix of p-values ${{\varvec{\Omega}}}$ under the null hypothesis. Since the exact correlations among all $L$ gene-based association tests are unknown, we perform the estimation procedure with $B$ replications. For each replicate $b$, $b = 1, \ldots ,B$, we implement the following two steps:

Step 1: We first generate a new Z-score vector ${\varvec{Z}}^{null}$ under the null hypothesis. That is, ${\varvec{Z}}^{null}$ follows a multivariate normal distribution with mean ${\varvec{0}}$ and variance–covariance matrix ${\mathbf{R}}$, where ${\mathbf{R}}$ can be estimated by LD among the genetic variants in a gene using external reference panels (i.e., 1000 Genomes Project).

Step 2: We use the regularization procedure to obtain the corrected correlation matrix of Z-scores ${\hat{\mathbf{U}}}$. Then, we calculate $Q_{BT}^{k\left( b \right)} ,Q_{SKAT}^{k\left( b \right)} ,Q_{SKATO}^{k\left( b \right)}$ and the corresponding p-values $p_{BT}^{k\left( b \right)} ,p_{SKAT}^{k\left( b \right)} ,p_{SKATO}^{k\left( b \right)}$ using the simulated ${\varvec{Z}}^{null}$ for $k = 0,1, \ldots ,K$. The distributions of $Q_{BT}^{k\left( b \right)} ,Q_{SKAT}^{k\left( b \right)} ,Q_{SKATO}^{k\left( b \right)}$ depend on the corrected correlation matrix ${\hat{\mathbf{U}}}$, and the standardized eQTL—derived weights ${\mathbf{W}}_{k}$ for $k = 0,1, \ldots ,K$.

To estimate the correlation matrix of p-values ${{\varvec{\Omega}}}$ used in the Overall method, we use the sample correlation matrix of the p-values obtained from the replications. We denote the sample correlation matrix of p-values as ${\hat{\mathbf{\Omega }}}$. For example, $\hat{\Omega }_{12}$ is the (1,2)-element of ${\hat{\mathbf{\Omega }}}$ which is the estimated correlation between BT and SKAT without using any weights. If we let ${\varvec{p}}_{BT}^{0} = \left( {p_{BT}^{0\left( 1 \right)} , \ldots ,p_{BT}^{0\left( B \right)} } \right)^{T}$ be a $B \times 1$ vector of the p-values of BT without using any weights and ${\varvec{p}}_{SKAT}^{0} = \left( {p_{SKAT}^{0\left( 1 \right)} , \ldots ,p_{SKAT}^{0\left( B \right)} } \right)^{T}$ be a $B \times 1$ vector of the p-values of SKAT without using any weights obtained from the replications, then the sample correlation of p-values between these two tests is defined as $\hat{\Omega }_{12} = {\text{cor}} \left( {{\varvec{p}}_{BT}^{0} ,{\varvec{p}}_{SKAT}^{0} } \right)$, where ${\text{cor}} ( \cdot )$ is the sample correlation.

The estimation procedure to estimate ${{\varvec{\Omega}}}$ is independent of our proposed method, therefore we only need to perform this procedure once for each gene. After we estimate ${{\varvec{\Omega}}}$, the p-value of Overall can be computed analytically without using permutations.

## Simulation studies

### Materials and comparison methods

In our studies, we use four data sets to obtain the eQTL—derived weights downloaded from the functional summary-based imputation website (http://gusevlab.org/projects/fusion/#reference-functionaldata). The resources to obtain the four eQTL—derived weights are listed in Table 1. For each eQTL data set, we use the weights estimated by the Best Linear Unbiased Prediction (BLUP)^27^.

**Table 1:** Resources of the four eQTL—derived weights used in the simulation studies.

Study	Tissue	# of samples	References
*NTR*	Peripheral blood	1247	Wright et al.^28^
*YFS*	Whole blood	1264	Gusev et al.^12^
*METSIM*	Adipose	563	Gusev et al.^12^
*CMC*	Brain	452	Gusev et al.^12^

We compare our proposed method with three existing methods, OT^15^, S-PrediXcan^29^, and S-TWAS^12^. These three methods are all based on GWAS summary statistics and incorporate eQTL‐derived weights. Here, we briefly introduce these three methods.

OT: For a total of $K$ different eQTL—derived weights and the three gene-based association tests (BT, SKAT, SKATO), OT aggregates information from different weights and tests by using the Cauchy combination method^30^. The test statistic of OT is defined as $Q_{OT} = \frac{1}{{3\left( {K + 1} \right)}}\sum\nolimits_{k = 0}^{K} {\left[ {\tan \left\{ {\left( {0.5 - p_{BT}^{k} } \right)\pi } \right\} + \tan \left\{ {\left( {0.5 - p_{SKAT}^{k} } \right)\pi } \right\} + \tan \left\{ {\left( {0.5 - p_{SKATO}^{k} } \right)\pi } \right\}} \right]}$ and the corresponding p-value of the test statistic can be approximated by $p_{OT} = \frac{1}{2} - \frac{{\arctan \left( {Q_{OT} } \right)}}{\pi }$.

S-PrediXcan: For a given eQTL‐derived weight, provided by a matrix ${\mathbf{W}}_{k} = {\text{diag}} \left( {W_{1}^{k} , \ldots ,W_{M}^{k} } \right)$, the test statistic of S-PrediXcan is defined as $Z_{S - PrediXcan}^{k} = \sum\nolimits_{m} {{{W_{m}^{k} \hat{\sigma }_{m} Z_{m} } \mathord{\left/ {\vphantom {{W_{m}^{k} \hat{\sigma }_{m} Z_{m} } {\hat{\sigma }}}} \right. \kern-\nulldelimiterspace} {\hat{\sigma }}}}$, where $\hat{\sigma }_{m}$ is the estimated standard deviation of the $m^{th}$ SNP in a gene and $\hat{\sigma }$ is the estimated standard deviation of the predicted expression of a gene. The p-value of S-PrediXcan can be computed as $p_{S - PrediXcan}^{k} = 2\Phi \left( { - \left| {Z_{S - PrediXcan}^{k} } \right|} \right)$, where $\Phi ( \cdot )$ is the standard normal CDF function.

S-TWAS: For a given eQTL‐derived weight, provided by a vector ${\varvec{w}}_{k} = \left( {W_{1}^{k} , \ldots ,W_{M}^{k} } \right)^{T}$, the test statistic of S-TWAS is defined as $Z_{S - TWAS}^{k} = \frac{{{\varvec{w}}_{k}^{T} \cdot {\varvec{Z}}}}{{\sqrt {{\varvec{w}}_{k}^{T} \cdot {\mathbf{R}} \cdot {\varvec{w}}_{k} } }}$, where ${\mathbf{R}}$ is the estimated LD structure among the genetic variants in a gene and the corresponding p-value can be calculated by $p_{S - TWAS}^{k} = 2\Phi \left( { - \left| {Z_{S - TWAS}^{k} } \right|} \right)$.

### The number of replications needed in estimation of ${{\varvec{\Omega}}}$

To apply our proposed method, we first need to estimate the correlation matrix of p-values, ${{\varvec{\Omega}}}$, under the null hypothesis for each gene. Following the estimation procedure introduced in the method section, we generate Z-scores instead of generating individual-level genotype and phenotype data. To determine the number of replications needed in the estimation of ${{\varvec{\Omega}}}$, we consider 18 genes that contain different numbers of SNPs and have different LD structures. Supplementary Table S1 gives a summary of these 18 genes. We can see from Supplementary Table S1, the number of SNPs in a gene is ranging from 23 to 359 and the average per-SNP LD score in a gene is ranging from 12.72 to 170.85. We simulate a Z-score vector from a multivariate normal distribution with mean ${\varvec{0}}$ and variance–covariance matrix ${\mathbf{R}}$, ${\varvec{Z}} \sim {\text{MVN}}\left( {{\varvec{0}},{\mathbf{R}}} \right)$, where ${\mathbf{R}}$ is the LD matrix of each gene which can be estimated using the 1000 Genomes Project (unrelated Europeans in 1000 Genomes in Phase 3; ftp://ftp.1000genomes.ebi.ac.uk/vol1/ftp/). First, we use $B = 10^{4}$ replications to estimate ${{\varvec{\Omega}}}$ under the null hypothesis, where the estimated matrix is denoted by ${\hat{\mathbf{\Omega }}}$. Then, we denote ${\hat{\mathbf{\Omega }}}^{0}$ as the correlation matrix of p-values by using $B_{0}$ replications. We vary the value of $B_{0}$ from 16 to 5000, and test the null hypothesis that the two correlation matrices, ${\hat{\mathbf{\Omega }}}^{0}$ and ${\hat{\mathbf{\Omega }}}$, are the same by using “lavaan” package (https://CRAN.R-project.org/package=lavaan)^31^. Supplementary Figure S1 shows that the p-values for the hypothesis testing in each gene are greater than 0.05 after $B_{0} = 1000$ replications for all of the 18 genes. Therefore, we recommend using 1000 replications to obtain ${\hat{\mathbf{\Omega }}}$ for each gene under the null hypothesis. Consequently, 1000 replications are used in the following sessions to evaluate the type I error rates and powers of Overall.

### Type I error rates

To evaluate if our proposed method can control type I error rates, we perform simulations based on the aforementioned 18 genes. For each of the 18 genes, we generate Z-score vectors under the null hypothesis, ${\varvec{Z}} \sim {\text{MVN}} \left( {{\varvec{0}},{\mathbf{R}}} \right)$, where ${\mathbf{R}}$ is the LD matrix of the gene estimated using the 1000 Genomes project. Then, we use the regularization procedure to obtain the corrected correlation matrix of Z-scores ${\hat{\mathbf{U}}}$, and calculate the three types of gene-based association tests, BT, SKAT, and SKATO, with or without the four eQTL—derived weights (NTR, YFS, METSIM, CMC) based on the corrected correlation matrix ${\hat{\mathbf{U}}}$. Finally, we apply our proposed method to combine the p-values using the estimated correlation matrix of p-values, ${\hat{\mathbf{\Omega }}}$, with 1000 replications.

We generate simulated data to mimic real lipids data which we will use in “Real data analysis” section. Gene *AGTRAP* is associated with lipids trait HDL^15^, There are a total of 23 genetic variants in gene *AGTRAP.* The LD block structure of these 23 genetic variants is shown in Supplementary Fig. S2. Supplementary Figure S3 shows the estimated correlation matrix ${\hat{\mathbf{\Omega }}}$ for this gene. We use $10^{7}$ replications to evaluate type I error rates of Overall for gene *AGTRAP* at $5 \times 10^{ - 2}$, $1 \times 10^{ - 2}$, $1 \times 10^{ - 3}$, $1 \times 10^{ - 4}$,$1 \times 10^{ - 5}$, and $1.75 \times 10^{ - 6}$ significance levels. With $10^{7}$ replications, a Bonferroni corrected significance level of $1.75 \times 10^{ - 6}$ can be reached to obtain the empirical type I error rates (i.e., for 28,625 genes in the real data analysis section, the Bonferroni corrected significance level is $0.05/28625 = 1.75 \times 10^{ - 6}$ at 5% significance level). We further evaluate type I rates based on the other 17 genes. To save computational time, we use $2 \times 10^{5}$ replications to evaluate type I error rates of Overall for the 17 genes at significance levels of $1 \times 10^{ - 2}$, $1 \times 10^{ - 3}$, and $1 \times 10^{ - 4}$. Table 2 and Supplementary Table S2 show the estimated type I error rates of Overall under various nominal significance levels for gene *AGTRAP *and the other 17 genes, respectively. From these tables, we can see that our proposed method can control type I error rates very well at different significant levels.

**Table 2:** Estimated type I error rates at different significance levels with $10^{7}$ replications. The subscript denotes BT, SKAT, and SKATO using eQTL—derived weights; CMC, METSIM, NTR, and YFS indicate the resources to obtain the eQTL—derived weights. 0 indicates the methods without using eQTL—derived weights.

$\alpha$-level	$5 \times 10^{ - 2}$	$1 \times 10^{ - 2}$	$1 \times 10^{ - 3}$	$1 \times 10^{ - 4}$	$1 \times 10^{ - 5}$	$1.75 \times 10^{ - 6}$
BT_0_	$5.03 \times 10^{ - 2}$	$1.06 \times 10^{ - 2}$	$1.00 \times 10^{ - 3}$	$1.01 \times 10^{ - 4}$	$9.76 \times 10^{ - 6}$	$1.84 \times 10^{ - 6}$
SKAT_0_	$5.24 \times 10^{ - 2}$	$1.07 \times 10^{ - 2}$	$1.01 \times 10^{ - 3}$	$1.00 \times 10^{ - 4}$	$1.04 \times 10^{ - 5}$	$1.80 \times 10^{ - 6}$
SKATO_0_	$4.58 \times 10^{ - 2}$	$9.57 \times 10^{ - 3}$	$1.02 \times 10^{ - 3}$	$1.04 \times 10^{ - 4}$	$9.72 \times 10^{ - 6}$	$1.46 \times 10^{ - 6}$
BT_CMC_	$5.17 \times 10^{ - 2}$	$1.04 \times 10^{ - 2}$	$1.01 \times 10^{ - 3}$	$9.82 \times 10^{ - 5}$	$9.58 \times 10^{ - 6}$	$1.72 \times 10^{ - 6}$
SKAT_CMC_	$5.08 \times 10^{ - 2}$	$9.89 \times 10^{ - 3}$	$9.71 \times 10^{ - 4}$	$9.75 \times 10^{ - 5}$	$9.48 \times 10^{ - 6}$	$1.66 \times 10^{ - 6}$
SKATO_CMC_	$5.16 \times 10^{ - 2}$	$1.09 \times 10^{ - 2}$	$1.17 \times 10^{ - 3}$	$1.21 \times 10^{ - 4}$	$1.22 \times 10^{ - 5}$	$2.14 \times 10^{ - 6}$
BT_METSIM_	$5.02 \times 10^{ - 2}$	$1.03 \times 10^{ - 2}$	$1.02 \times 10^{ - 3}$	$1.01 \times 10^{ - 4}$	$9.86 \times 10^{ - 6}$	$1.66 \times 10^{ - 6}$
SKAT_METSIM_	$5.30 \times 10^{ - 2}$	$1.08 \times 10^{ - 2}$	$1.02 \times 10^{ - 3}$	$9.91 \times 10^{ - 5}$	$1.00 \times 10^{ - 5}$	$2.12 \times 10^{ - 6}$
SKATO_METSIM_	$4.84 \times 10^{ - 2}$	$1.05 \times 10^{ - 2}$	$1.11 \times 10^{ - 3}$	$1.09 \times 10^{ - 4}$	$1.06 \times 10^{ - 5}$	$1.84 \times 10^{ - 6}$
BT_NTR_	$5.02 \times 10^{ - 2}$	$1.06 \times 10^{ - 2}$	$1.00 \times 10^{ - 3}$	$9.93 \times 10^{ - 5}$	$1.01 \times 10^{ - 5}$	$1.76 \times 10^{ - 6}$
SKAT_NTR_	$5.09 \times 10^{ - 2}$	$1.03 \times 10^{ - 2}$	$9.98 \times 10^{ - 4}$	$1.00 \times 10^{ - 4}$	$1.01 \times 10^{ - 5}$	$2.00 \times 10^{ - 6}$
SKATO_NTR_	$5.08 \times 10^{ - 2}$	$1.18 \times 10^{ - 2}$	$1.34 \times 10^{ - 3}$	$1.45 \times 10^{ - 4}$	$1.52 \times 10^{ - 5}$	$2.92 \times 10^{ - 6}$
BT_YFS_	$5.10 \times 10^{ - 2}$	$1.02 \times 10^{ - 2}$	$9.95 \times 10^{ - 4}$	$9.95 \times 10^{ - 5}$	$1.05 \times 10^{ - 5}$	$2.10 \times 10^{ - 6}$
SKAT_YFS_	$4.98 \times 10^{ - 2}$	$1.03 \times 10^{ - 2}$	$9.97 \times 10^{ - 4}$	$1.01 \times 10^{ - 4}$	$1.02 \times 10^{ - 5}$	$2.06 \times 10^{ - 6}$
SKATO_YFS_	$5.58 \times 10^{ - 2}$	$1.32 \times 10^{ - 2}$	$1.43 \times 10^{ - 3}$	$1.55 \times 10^{ - 4}$	$1.69 \times 10^{ - 5}$	$3.50 \times 10^{ - 6}$
Overall	$4.67 \times 10^{ - 2}$	$1.01 \times 10^{ - 2}$	$1.12 \times 10^{ - 3}$	$1.14 \times 10^{ - 4}$	$1.24 \times 10^{ - 5}$	$2.44 \times 10^{ - 6}$

### Power comparison

To evaluate the performance of the Overall method, we use several simulations to compare the power of Overall with the power of OT, S-PrediXcan, S-TWAS, and three types of gene-based association tests with and without eQTL—derived weights. We use BEST to represent the test with the maximum power among the three traditional gene-based association tests with and without an eQTL—derived weight, S-TWAS.B and S-PrediXcan.B to represent the maximum power of S-TWAS and S-PrediXcan with each of the eQTL—derived weights, respectively. Following the simulation settings in Nagpal et al.^32^ and Zhang et al.^15^, we generate individual-level genotypes, phenotypes, and different gene expression levels using the following steps:

1. The genotype data are generated using the haplotypes of a gene obtained from the 1000 Genomes Project reference panel. To generate the genotype of an individual, ${\mathbf{X}}_{g}$, we select two haplotypes according to the haplotype frequencies from the haplotype pool and then remove genetic variants with MAF < 0.05.
2. We consider $K$ different weights derived from gene expression data which can be estimated using BLUP. To generate a vector of weights, ${\varvec{w}}_{k}$, for the *k*th gene expression level, we randomly select causal variants according to the proportion of causal variants, $p_{causal}$. Then, the effect sizes for the *k*th gene expression levels and $M_{causal}$ causal variants can be generated from a standard normal distribution, $w_{mk} \sim N\left( {0,1} \right)$ for $m = 1, \ldots ,M_{causal}$, where $M_{causal} = M \times p_{causal}$; otherwise, $w_{mk} = 0$. After we rescaled the weights to ensure the targeted expression heritability $h_{e}^{2}$, we generate the *k*th gene expression level by $\user2{E}_{k} = {\mathbf{X}}_{g} \user2{w}_{k} + {\varvec{\varepsilon}}_{e}$ with each element of random error ${\varvec{\varepsilon}}_{e}$ follows $N\left( {0,1 - h_{e}^{2} } \right)$.
3. Let ${\textbf{E}} = \left( {\boldsymbol{E}_{1} , \ldots ,\boldsymbol{E}_{K} } \right)$ be the matrix of gene expression levels. Phenotypes are generated by using a formula $\user2{Y} = {\mathbf{E}}\varvec{\beta } +{\varvec{\varepsilon}}_{p}$ with each element of random error ${\varvec{\varepsilon}}_{p}$ follows $N\left( {0,1 - h_{p}^{2} } \right)$, where ${\varvec{\beta}} = \left( {\beta_{1} , \ldots ,\beta_{K} } \right)^{T}$ is a vector of genetic effect sizes which can be assigned based on the phenotypic heritability $h_{p}^{2}$.
4. The Z-score vector is estimated from individual-level genotype and phenotype data using beta coefficient and its standard deviation estimated based on the ordinary least squares method in linear regression.

In our simulation studies for power comparison, we consider two genes, *AGTRAP* and *C3orf22,* from the 18 genes used in the type I error evaluation and $K = 4$ and $K = 20$ eQTL—derived weights. *AGTRAP* contains 458 haplotypes for 23 genetic variants (11 common variants and 12 rare variants; MAF ranging from 0 to 0.39775); *C3orf22* contains 295 haplotypes for 42 variants (18 common variants and 24 rare variants; MAF ranging from 0 to 0.43558). Supplementary Figure S2 shows the LD block structure of the 23 genetic variants at *AGTRAP* and the 42 genetic variants at *C3orf22*. We vary the proportion of causal variants with values $p_{causal} = (0.2,0.3,0.4,0.5)$ for *AGTRAP* and $p_{causal} = (0.1,0.2,0.3,0.4)$ for *C3orf22*. We also consider two different directions of genetic effects: $\beta_{1} = \cdots = \beta_{K}$ (Scenario 1: Uni-directional effects) and $\beta_{1} = \cdots = \beta_{K/2} = - \beta_{K/2 + 1} = \cdots = - \beta_{K}$ (Scenario 2: Bi-directional effects). For each simulation scenario, we vary the proportion of gene expression heritability and the phenotypic heritability with different values of $h_{e}^{2}$ and $h_{p}^{2}$. We consider the sample size to be 2000 (unless it is specified) and the power is calculated as the proportion of 1000 replications with p-value $< 1.75 \times 10^{ - 6}$.

**Figure 1:**
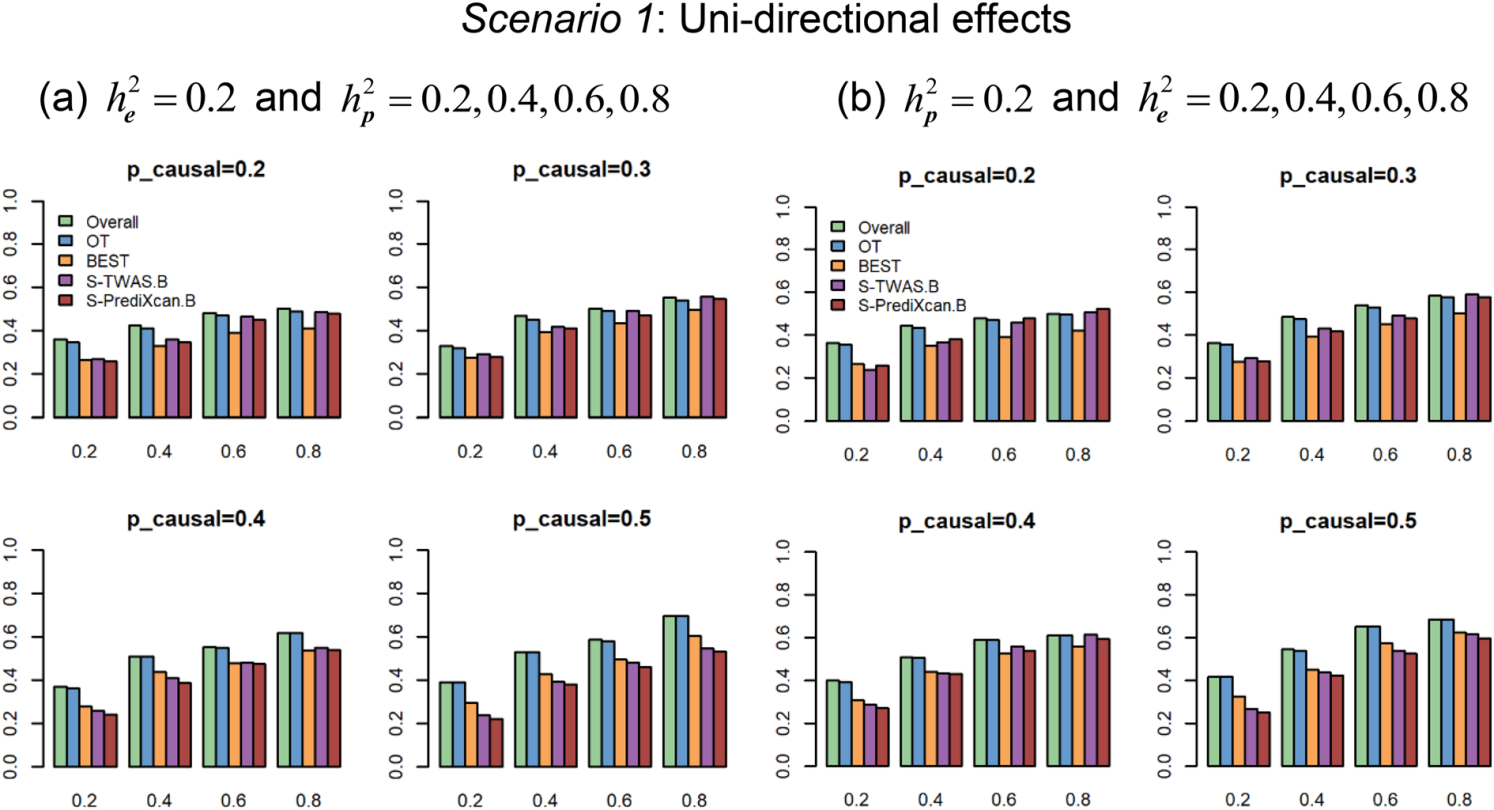
Power comparisons of gene-based association tests at $1.75 \times 10^{ - 6}$ significance level under Uni-directional effects ($\beta_{1} = \beta_{2} = \beta_{3} = \beta_{4}$) with $p_{causal} = \left( {0.2,0.3,0.4,0.5} \right)$ based on gene *AGTRAP*. (**a**) Estimated power against phenotypic heritability $h_{p}^{2}$ with fixed expression heritability $h_{e}^{2} = 0.2$; (**b**) Estimated power against expression heritability $h_{e}^{2}$ with fixed phenotypic heritability $h_{p}^{2} = 0.2$.

**Figure 2:**
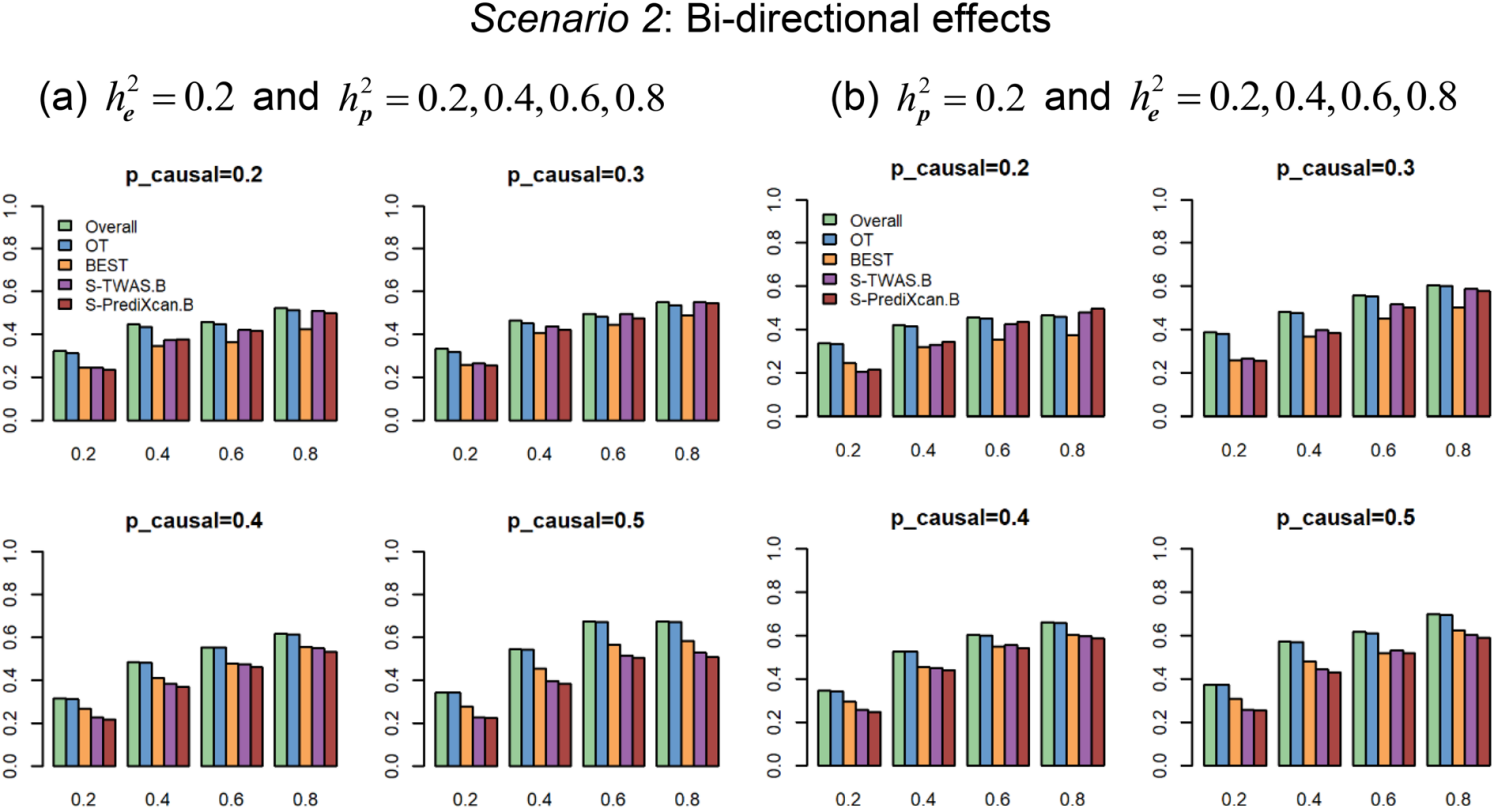
Power comparisons of gene-based association tests at $1.75 \times 10^{ - 6}$ significance level under Bi-directional effects ($\beta_{1} = \beta_{2} = - \beta_{3} = - \beta_{4}$) with $p_{causal} = \left( {0.2,0.3,0.4,0.5} \right)$ based on gene *AGTRAP*. (**a**) Estimated power against phenotypic heritability $h_{p}^{2}$ with expression heritability $h_{e}^{2} = 0.2$; (**b**) Estimated power against expression heritability $h_{e}^{2}$ with phenotypic heritability $h_{p}^{2} = 0.2$.

Figure 1 (Supplementary Fig. S4) show the power comparisons based on gene *AGTRAP* (and *C3orf22*) with $K = 4$ under the Uni-directional effects ($\beta_{1} = \beta_{2} = \beta_{3} = \beta_{4}$) with different $p_{causal}$. We consider two settings here. First, we vary phenotypic heritability $h_{p}^{2}$ with a fixed expression heritability $h_{e}^{2} = 0.2$ (Fig. 1a and Supplementary Fig. S4a). Second, we vary the expression heritability $h_{e}^{2}$ with a fixed phenotypic heritability $h_{p}^{2} = 0.2$ (Fig. 1b and Supplementary Fig. S4b). Figure 2 (Supplementary Fig. S5) shows power comparisons based on gene *AGTRAP* (and *C3orf22*) under the Bi-directional effects ($\beta_{1} = \beta_{2} = - \beta_{3} = - \beta_{4}$) with different $p_{causal}$ for $K = 4$. We also consider two simulation settings, power against the phenotypic heritability $h_{p}^{2}$ with a fixed expression heritability $h_{e}^{2} = 0.2$ and power against the expression heritability $h_{e}^{2}$ with a fixed phenotypic heritability $h_{p}^{2} = 0.2$. The pattern of the power in Fig. 2 (Supplementary Fig. S5) is similar to what we observe in Fig. 1 (Supplementary Fig. S4). These figures show that (1) Overall and OT perform uniformly better than BEST, S-TWAS.B, and S-PrediXcan.B. We can see that Overall and OT boost power significantly due to integrating association evidence by different traditional tests and multiple eQTL—derived weights. Overall is slightly more powerful than OT in all of the scenarios. (2) Among BEST, S-TWAS.B, and S-PrediXcan.B, BEST is more powerful than S-TWAS.B and S-PrediXcan.B in all of the scenarios for gene *C3orf22*; For gene *AGTRAP*, S-TWAS.B and S-PrediXcan.B perform better than BEST when the proportion of causal variants in a gene is small ($p_{causal} = (0.2,0.3)$); otherwise, BEST performs better than S-TWAS.B and S-PrediXcan.B.

To evaluate if Overall and OT that integrate different types of association tests and multiple eQTL—derived weights are robust for more eQTL studies, we also consider 20 ($K = 20$) eQTL—derived weights under Uni-directional effect and Bi-directional effect models on gene *C3orf22* with settings similar to the settings in Supplementary Figs. S4 and S5. After integrating $L = 3\left( {K + 1} \right) = 63$ traditional gene-based association tests, we observe that the patterns of the power for $K = 20$ are similar to that in Supplementary Figs. S4 and S5 with $K = 4$, and the power gain of Overall and OT is higher than that of the tests only consider one eQTL—derived weight, such as BEST, S-PrediXcan.B, and S-TWAS.B (Supplementary Fig. S6).

Furthermore, we consider simulation settings with noise to the eQTL. We consider simulation settings by adding less noise to the eQTL from the most relevant tissues and more noise to those from the less relevant tissues. For the Uni-direction scenario, we consider the first study being the most relevant tissue, where $\beta_{1} = \beta_{0} + N\left( {0,0.1h_{p}^{2} } \right)$ and $\beta_{2} = \beta_{3} = \beta_{4} = \beta_{0} + N\left( {0,0.5h_{p}^{2} } \right)$; $\beta_{0} = \sqrt {{{h_{p}^{2} } \mathord{\left/ {\vphantom {{h_{p}^{2} } K}} \right. \kern-\nulldelimiterspace} K}}$ depends on the phenotypic heritability $h_{p}^{2}$. For the Bi-direction scenario, we consider the first and third studies being the most relevant tissues that have opposite effect directions, where $\beta_{1} = - \beta_{0} + N\left( {0,0.1h_{p}^{2} } \right), \, \beta_{3} = \beta_{0} + N\left( {0,0.1h_{p}^{2} } \right),$ and $\beta_{2} = - \beta_{0} + N\left( {0,0.5h_{p}^{2} } \right), \, \beta_{4} = \beta_{0} + N\left( {0,0.5h_{p}^{2} } \right)$. Other parameter settings are the same as these in Supplementary Figs. S4 and S5. The power comparison results are shown in Supplementary Figs. S7 and S8. From these figures, we find that the patterns of the power in Supplementary Figs. S7 and S8 are very similar to those in Supplementary Figs. S4 and S5.

In all of the previous power comparisons, we use a sample size of 2000. We also consider simulation settings as those in Supplementary Figs. S7 and S8, but with a large sample size of 100,000. Supplementary Figure S9 shows the results of power comparisons. We can see from this figure, all powers are increased with this larger sample size, but the patterns of the power are very similar to those in Supplementary Figs. S7 and S8.

To remove noise in LD matrix computed from a reference sample, we shrink the observed LD matrix toward an identity matrix with the shrinkage parameter estimated by maximum likelihood. To evaluate how well this regulation process performs, we compare the powers of three traditional gene-based association tests with and without eQTL—derived weights, OT, and Overall based on corrected and uncorrected LD structure. We use the same simulation settings as those in Supplementary Figs. S7 and S8. Supplementary Figure S10 shows the power comparison results based on gene *C3orf22* under Uni-directional effects and Bi-directional effects with noise to eQTL. We can see that the powers of these tests based on corrected LD structure perform better than those based on uncorrected LD structure in most of the settings.

## Real data analysis

To evaluate the performance of our proposed method, we apply Overall, OT, the three traditional tests with and without eQTL—derived weights, S-PrediXcan, and S-TWAS to the GWAS summary statistics data sets used in Zhang et al.^15^: two SCZ GWAS summary data sets and two lipid GWAS summary data sets. We estimate the LD between genetic variants using the 1000 Genomes Project reference panel^16^, and obtain the corrected matrix of Z-score after the regularization procedure. We consider four eQTL—derived weights estimated by the BLUP method using the resources listed in Table 1 (NTR, YFS, METSIM, CMC).

### Application to the SCZ GWAS summary data

We consider two SCZ GWAS summary data sets, SCZ1 and SCZ2, which can be downloaded from the Psychiatric Genomics Consortium website (https://www.med.unc.edu/pgc/results‐and‐downloads/)^33^. SCZ1 is a meta-analysis of SCZ GWAS data set with 13,833 cases and 18,310 controls. SCZ2 is a more recent and larger SCZ GWAS summary data set with 36,989 cases and 113,075 controls for partial validation^34^. In our real data analysis, we define a gene to include all of the SNPs from 20 kb upstream to 20 kb downstream of the gene and test the association between each gene and the trait. We consider all genes according to the GENCODE version 35 (GRCh37) human comprehensive gene annotation list which can be downloaded from the GENCODE website (https://www.gencodegenes.org/human/release_35lift37.html).

**Figure 3:**
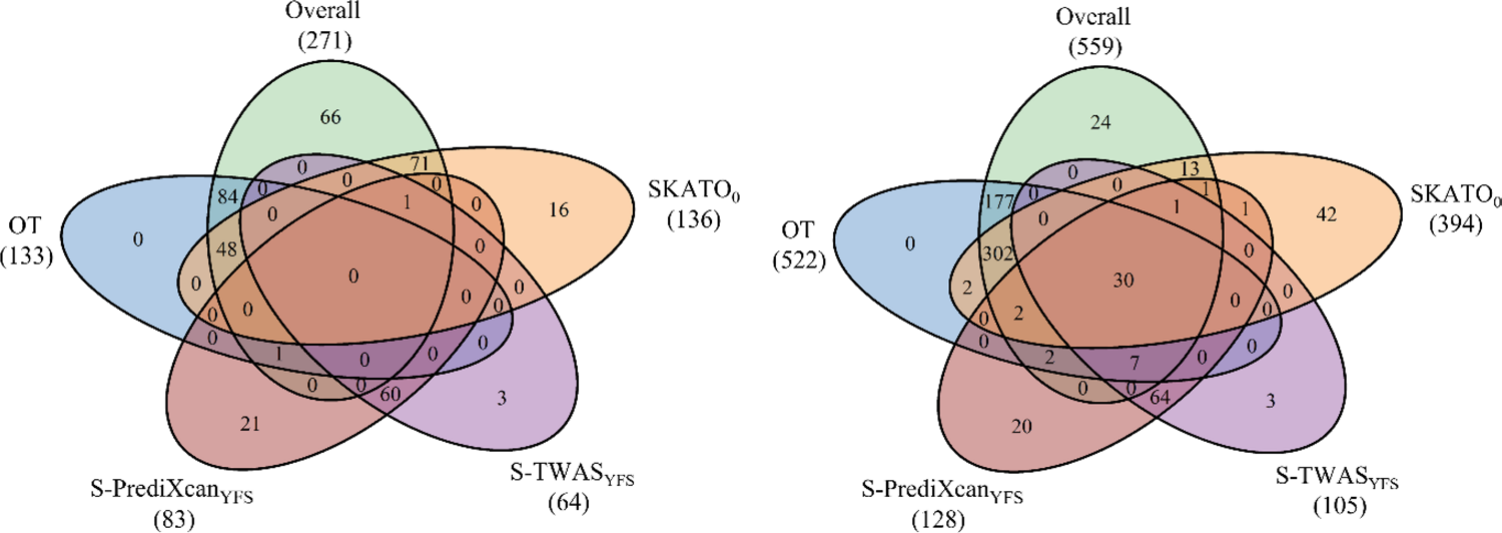
Venn diagram of the number of genes identified by Overall, OT, and SKATO_0_, S-PrediXcan_YFS_, and S-TWAS_YFS_ for SCZ1 data (left) and SCZ2 data (right). The number below each method indicates the total number of significant genes identified by the corresponding method.

To make fair comparisons among all these weighted tests, the genetic variants are removed if there is at least one weight missing in the four eQTL—derived weights. After pruning, there are 26,575 genes in SCZ1 and 17,823 genes in SCZ2 left in our final analyses. Therefore, the Bonferroni corrected significance level for gene-based association analysis is defined as 0.05 divided by the number of genes. First, we apply BT, SKAT, and SKATO with and without an eQTL—derived weight, OT, Overall, S-PrediXcan, and S-TWAS to the SCZ1 and SCZ2 data sets. Table 3 (SCZ1 and SCZ2) shows the number of genes identified by each method for the SCZ data sets, respectively. As we can see in Table 3, Overall identifies more genes than all of the other methods for two SCZ GWAS summary data sets. Among the three types of gene-based association tests, BT, SKAT, and SKATO, with or without different eQTL—derived weights, SKATO_0_ identifies the largest number of genes. S-TWAS_YFS_ and S-PrediXcan_YFS_ identify the largest number of genes compared with S-TWAS and PrediXcan based on the other three eQTL—derived weights, respectively. Therefore, in Fig. 3, we only show the number of genes identified by Overall, OT, SKATO_0_, S-PrediXcan_YFS_, and S-TWAS_YFS_. The number below each method indicates the total number of genes identified by the corresponding method. From Fig. 3, we can see that Overall identifies all of the genes identified by OT for SCZ1; for SCZ2, there are two genes identified by OT but failed to be identified by Overall; there are 66 and 24 genes identified only by Overall for SCZ1 data and SCZ2, respectively.

**Table 3:** The numbers of genes identified by each method for the two SCZ data sets. The subscript denotes BT, SKAT, and SKATO using eQTL—derived weights; CMC, METSIM, NTR, and YFS indicate the resources to obtain the eQTL—derived weights. 0 indicates the methods without using any weights.

	SCZ1	SCZ2	SCZ_overlap_	GWAS_SCZ1_	GWAS_SCZ2_
BT_0_	97	166	7	1	38
SKAT_0_	47	305	20	15	153
SKATO_0_	136	394	27	15	153
BT_CMC_	44	137	2	1	56
SKAT_CMC_	12	225	6	1	134
SKATO_CMC_	30	263	2	1	130
BT_METSIM_	44	136	5	1	48
SKAT_METSIM_	23	223	9	4	132
SKATO_METSIM_	31	205	3	0	100
BT_NTR_	48	119	7	6	48
SKAT_NTR_	27	230	9	8	141
SKATO_NTR_	40	280	8	6	143
BT_YFS_	89	166	14	1	53
SKAT_YFS_	20	223	6	7	137
SKATO_YFS_	47	321	7	0	140
S-PrediXcan_CMC_	42	43	7	0	38
S-PrediXcan_METSIM_	41	44	8	1	30
S-PrediXcan_NTR_	48	70	14	6	59
S-PrediXcan_YFS_	83	128	29	2	72
S-TWAS_CMC_	33	45	6	0	43
S-TWAS_METSIM_	36	29	5	1	20
S-TWAS_NTR_	37	54	13	6	46
S-TWAS_YFS_	64	105	29	2	58
OT	133	522	17	6	166
Overall	271	559	45	16	167

SCZ1 indicates the number of genes identified by each method for SCZ1 data; SCZ2 indicates the number of genes identified by each method for SCZ2 data; SCZ_overall_ indicates the number of overlapping genes identified by both SCZ1 and SCZ2 data sets; GWAS_SCZ1_ and GWAS_SCZ2_ indicate the numbers of genome-wide significant genes that are reported in the GWAS catalog and are also identified by each method for SCZ1 and SCZ2, respectively.

We further investigate the 90 genes identified only by Overall for the SCZ data sets by searching the GWAS catalog (https://www.ebi.ac.uk/gwas/). Among the 66 genes for the SCZ1 data set, there are six genes reported in the GWAS catalog; among the 24 genes for the SCZ2 data set, there are six genes reported in the GWAS catalog (Table 4). We also use these two SCZ GWAS data sets for partial validation. Table 3 shows that there are 45 overlapping genes identified by Overall using SCZ1 and SCZ2 data sets and only 17 overlapping genes identified by OT using both SCZ1 and SCZ2 data sets. Furthermore, we search for genome-wide significant SNPs ($p < 5 \times 10^{ - 8}$) from the two SCZ GWAS summary data sets and consider the genes covering at least one genome-wide significant SNP from 20 kb upstream to 20 kb downstream of the gene. There are 63 genome-wide significant genes for SCZ1, and 2422 genome-wide significant genes in SCZ2. Table 3 (GWAS_SCZ1_ and GWAS_SCZ2_) summarizes the numbers of genome-wide significant genes that are identified by each method for the two SCZ data sets. Among the 63 genome-wide significant genes for the SCZ1 data set, Overall identifies the largest number of genes, followed by SKAT_0_ and SKATO_0_; OT, S-PrediXcan_NTR_ and S-TWAS_NTR_ only identify 6 genes. Meanwhile, among 2422 genome-wide significant genes for SCZ2, Overall identifies 167 genes; OT identifies 166 genes; SKATO and SKATO_0_ identify 153 genes; S-TWAS_YFS_ and S-PrediXcan_YFS_ only identify 58 and 72 genes respectively.

**Table 4:** Genes identified only by Overall based on the two SCZ data sets that are reported in the GWAS catalog.

Gene	Data	Overall	References
*RAI1*	SCZ1	2.63E−31	Pardiñas et al.^35^
*SLC7A6*	SCZ1	2.17E−15	Ikeda et al.^36^; Li et al.^37^
*AP001931.2*	SCZ1	1.27E−13	Schizophrenia Working Group of the Psychiatric Genomics Consortium^34^; Goes et al.^38^; Ikeda et al.^36^; Li et al.^37^; Lam et al.^39^; Periyasamy et al.^40^; Lee et al.^41^; The Autism Spectrum Disorders Working Group of the Psychiatric Genomics Consortium^42^; Pardiñas et al.^35^
*MARK2*	SCZ1	2.64E−07	Goes et al.^38^
*GULOP*	SCZ1	1.24E−07	Pardiñas et al.^35^; Ikeda et al.^36^; Li et al.^37^; Goes et al.^38^; Lam et al.^43^
*ZBED4*	SCZ1	9.02E−07	Goes et al.^38^
*RAB11FIP5*	SCZ2	1.05E−06	Goes et al.^38^; Lam et al.^43^
*AL669918.1*	SCZ2	2.03E−06	Goes et al.^38^
*YPEL1*	SCZ2	2.80E−06	Goes et al.^38^
*LINC00606*	SCZ2	2.57E−06	Goes et al.^38^
*ERLIN1*	SCZ2	2.34E−06	Goes et al.^38^
*AC024597.1*	SCZ2	2.56E−06	Lam et al.^39^

### Application to the lipids GWAS summary data

**Figure 4:**
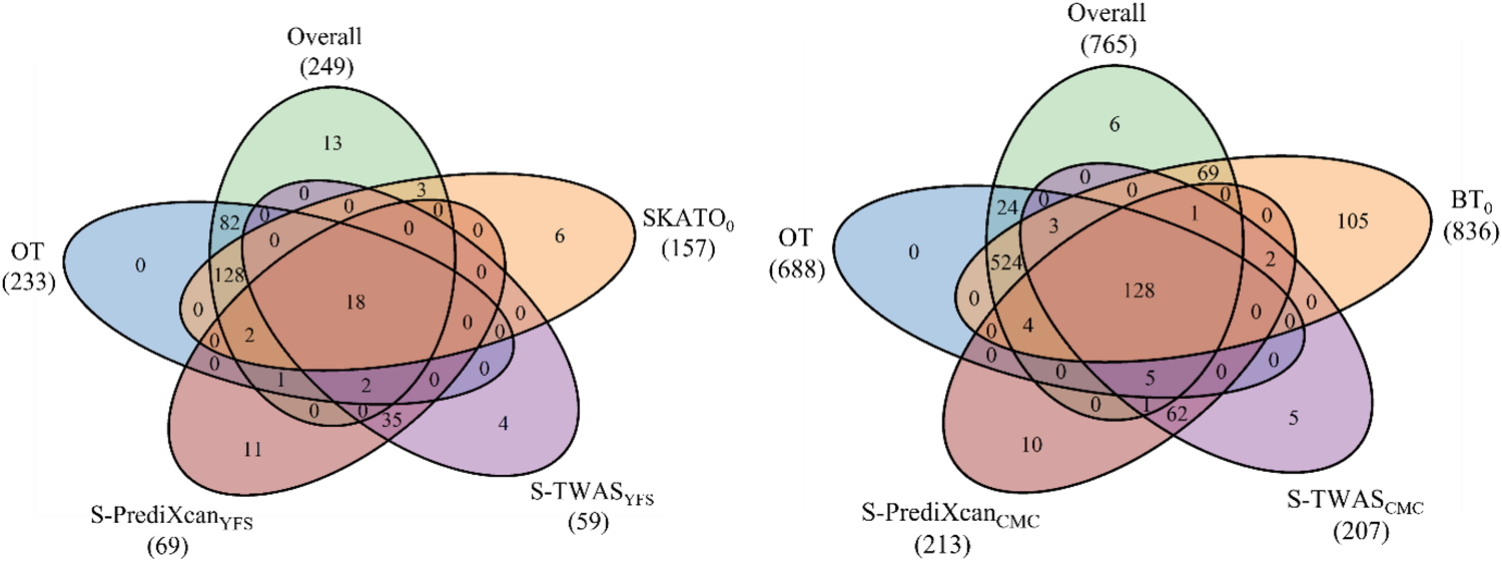
Venn diagram of the number of genes identified by Overall, OT, SKATO_0_, S-PrediXcan_YFS_, and S-TWAS_YFS_ for HDL1 data (left) and Overall, OT, BT_0_, S-PrediXcan_CMC_, and S-TWAS_CMC_ for HDL2 data (right). The number below each method indicates the total number of significant genes identified by the corresponding method.

We consider two lipids GWAS summary data sets, HDL1 and HDL2, which can be downloaded at the Center for Statistical Genetics (CSG) at the University of Michigan. HDL1 is a meta-analysis of HDL GWAS data set with about 100,000 samples downloaded at the website (http://csg.sph.umich.edu/willer/public/lipids2010/)^44^. HDL2 is the follow-up data with about 189,000 samples for partial validation downloaded at the Global Lipids Genetics Consortium (http://csg.sph.umich.edu/willer/public/lipids2013/)^45^. We perform the same analysis as we did in the previous section for the two SCZ GWAS summary data sets. After pruning and removing the genetic variants with missing weights, there are 17,389 genes in HDL1 and 16,917 genes in HDL2. Table 5 (HDL1 and HDL2) shows the number of genes identified by each method for the two lipids data sets, respectively. As we can see from Table 5, among the three traditional gene-based association tests with and without eQTL—derived weights, SKATO_0_ and BT_0_ identify the largest number of genes in HDL1 and HDL2, respectively; Among the four S-PrediXcan tests, S-PrediXcan_YFS_ and S-PrediXcan_CMC_ identify the largest number of genes in HDL1 and HDL2, respectively; for the four S-TWAS tests, S-TWAS_YFS_ and S-TWAS_CMC_ identify the largest number of genes in HDL1 and HDL2, respectively. For the HDL1 data set, Overall identifies the largest number of genes (249), followed by OT that identifies 233 genes; for the HDL2 data set, BT_0_ identifies the largest number of genes (836), followed by Overall and OT, where Overall identifies 765 genes and OT identifies 688 genes. In Fig. 4, we compare genes identified by SKATO_0_, S-PrediXcan_YFS_, and S-TWAS_YFS_, along with Overall and OT for the HDL1 data set and genes identified by BT_0_, S-PrediXcan_CMC_, S-TWAS_CMC_, Overall, and OT for the HDL2 data set. Again, we observe that Overall identifies the largest number of genes for the HDL1 data set and the second most for the HDL2 data set; all genes identified by OT are also identified by Overall; 82 and 24 genes are identified only by Overall and OT for the HDL1 and HDL2 data sets, respectively; there are 13 and 6 genes only identified by Overall for the HDL1 and HDL2 data sets, respectively. We search the GWAS catalog (https://www.ebi.ac.uk/gwas/). Table 6 shows that five out of 13 genes identified only by Overall based on HDL1 data have been reported, and one out of 6 genes has been reported on HDL2 data in the GWAS catalog. We also use these two HDL GWAS data sets for partial validation by looking for the number of overlapping genes identified by both of the data sets (Table 5, HDL_overlap_). There are 177 overlapping genes identified by Overall for both SCZ1 and SCZ2 data sets and 167 overlapping genes identified by OT for both SCZ1 and SCZ2 data sets.

**Table 5:** The number of genes identified by each method for the two lipids data sets. The subscript denotes BT, SKAT, and SKATO using eQTL—derived weights; CMC, METSIM, NTR, and YFS indicate the resources to obtain the eQTL—derived weights. 0 indicates the methods without using any weights.

	HDL1	HDL2	HDL_overlap_	GWAS_HDL1_	GWAS_HDL2_
BT_0_	95	836	78	50	185
SKAT_0_	116	174	114	99	157
SKATO_0_	157	762	138	104	190
BT_CMC_	79	130	41	46	107
SKAT_CMC_	105	159	99	95	146
SKATO_CMC_	130	177	103	96	150
BT_METSIM_	83	160	59	58	111
SKAT_METSIM_	120	259	118	102	149
SKATO_METSIM_	131	199	118	98	152
BT_NTR_	78	136	50	49	111
SKAT_NTR_	105	156	100	90	148
SKATO_NTR_	131	183	111	95	154
BT_YFS_	88	154	50	53	113
SKAT_YFS_	106	148	102	94	137
SKATO_YFS_	142	185	112	99	144
S-PrediXcan_CMC_	43	213	18	29	114
S-PrediXcan_METSIM_	45	201	23	30	118
S-PrediXcan_NTR_	33	187	14	19	108
S-PrediXcan_YFS_	69	195	25	31	117
S-TWAS_CMC_	40	207	17	23	109
S-TWAS_METSIM_	37	202	16	15	112
S-TWAS_NTR_	25	176	10	11	97
S-TWAS_YFS_	59	183	24	29	115
OT	233	688	167	120	190
Overall	249	765	177	122	192

HDL1 indicates the number of genes identified by each method for HDL1 data; HDL2 indicates the number of genes identified by each method for HDL2 data; HDL_overall_ indicates the number of overlapping genes identified by both HDL1 and HDL2 data sets; GWAS_HDL1_ and GWAS_HDL2_ indicate the numbers of genome-wide significant genes that are reported in the GWAS catalog and are also identified by each method for HDL1 and HDL2, respectively.

**Table 6:** Genes identified only by Overall based on the two lipids data sets that are reported in the GWAS catalog.

Gene	Data	Overall	References
*AP002954.1*	HDL1	2.27E−11	Emilsson et al.^46^
*EDC4*	HDL1	1.65E−11	Lettre et al.^47^, Kilpeläinen et al.^48^, Wojcik et al.^49^
*PACSIN1*	HDL1	2.24E−06	Liu et al.^50^
*AFF1*	HDL1	2.10E−06	Spracklen et al.^51^, De Vries et al.^52^, Hoffmann et al.^53^, Ripatti et al.^54^, Richardson et al.^55^
*AC106779.1*	HDL1	2.85E−06	Noordam et al.^56^
*NHLRC2*	HDL2	1.98E−06	Hoffmann et al.^53^, Richardson et al.^55^, Klarin et al.^57^, Qi et al.^58^, Klimentidis et al.^59^, Liu et al.^60^

Same as the analyses for the SCZ GWAS summary data sets, we search for genome-wide significant SNPs ($p < 5 \times 10^{ - 8}$) from the two lipids GWAS summary statistics. There are 1911 genome-wide significant genes for HDL1 and 2682 genome-wide significant genes for HDL2. Table 5 (GWAS_HDL1_ and GWAS_HDL2_) summarizes the numbers of genome-wide significant genes that are identified by each method for the two lipids data sets. Among the 1911 genome-wide significant genes for the HDL1 data set, Overall identifies the largest number of genes (122), followed by OT (120), then SKAT_0_ (104); S-TWAS_YFS_ only identifies 29 genes and S-PrediXcan_YFS_ identifies 31 genes. Meanwhile, among 2682 genome-wide significant genes for HDL2, Overall identifies the largest number of genes (192); OT and SKATO_0_ identify 190 genes; S-TWAS_METSIM_ and S-PrediXcan_METSIM_ identify 112 and 118 genes. respectively.

## Discussions

In this paper, we develop a powerful and computationally efficient method, Overall, for gene-based association studies using GWAS summary data. Overall aggregates information from three traditional types of gene-based association tests (BT, SKAT, SKATO) and also incorporates eQTL data. Both our simulation studies and real data analysis confirm that our proposed method can control type I error rates correctly and has very good performance compared with other comparison methods. In “Real data analysis”, Overall identify more significant genes than other methods, and there are some genes reported by GWAS catalog which are only identified by Overall.

There are some advantages of our proposed method. First, Overall adaptively aggregates information from multiple gene-based association tests. Most combination tests (i.e., Fisher’s combination test^61^) assume that the p-values should be calculated from independent tests. To combine information from highly correlated gene-based association tests, Overall utilizes the extended Simes procedure^5,22^. It is shown that this procedure to combine multiple tests is stable and effective regardless of whether the tests are highly correlated^24,62^. Second, Overall is more powerful than the traditional gene-based association tests, some popular transcriptome association tests (i.e., S-PrediXcan^29^ and S-TWAS^12^), and other eQTL weighted combination tests (i.e., ominous test^15^). By aggregating information from different tests and incorporating multiple eQTL—derived weights, Overall can achieve a higher statistical power under a variety of situation settings. Meanwhile, our simulation studies and real data analyses show that the extended Simes procedure is more powerful than the Cauchy combination method, especially if the proportion of causal variants in a gene is small. Third, the p-values of Overall can be analytically computed without using permutations, therefore, Overall is computationally efficient. Finally, using the regularization procedure to correct the estimated LD can reduce the potential statistical noise in the LD estimation if LD is estimated using a reference panel with small sample size. In addition, Overall can be easily applied to genetic association studies with either individual-level data or GWAS summary statistics.

In this paper, we combine three types of traditional gene-based association tests (BT, SKAT, SKATO). However, the combination procedure used in the paper is very general. Other more powerful gene-based association tests can also be combined using the same approach, such as some state-of-the-art methods (i.e., S-TWAS^12^, E-MAGMA^63^, and SMR^64^).

In this current study, we utilize the weights derived from four single tissue gene expression studies (CMC, METSIM, NTR, YFS). Although the extended Simes procedure in Overall allows us to employ more eQTL—derived weights from a number of studies (i.e., GTEx gene expression version 8^65^ et al.), there is a possibility that the noise can be increased with the increment in the number of unrelated studies. Therefore, the power of the combination tests (i.e., Overall and OT) might be attenuated. Thus, to obtain the most robust identification of phenotypic associated genes in a real data analysis with the Overall method, we suggest incorporating eQTL datasets from the most relevant tissues to the phenotype. The last but the most important thing is that population stratification can be confounded association results^66,67^. Systematic minor allele frequency difference between transcriptomic studies of different cohorts and no matching between the estimated LD structure of Genomes Project with that in the study may increase the chances of false positive findings. Therefore, we need to eliminate false positive findings possibly caused by population stratification^68,69^. When applying the Overall method, the population of GWAS summary dataset, external reference panel (i.e., 1000 Genomes Project) used to estimate LD structure, and eQTL—derived weights should be consistent.

In this study, the computational time of the proposed method is acceptable even if the estimated correlation matrix of multiple tests is obtained by the replication procedure. Meanwhile, the estimation procedure is independent of gene-based association tests, therefore we only need to perform this procedure once for each GWAS summary dataset. For example, there are a total of 29,008 gene in the 1000 Genomes Project and we use 1000 replicates to estimate the correlation matrix of multiple tests for each gene. We perform this using the high-performance computing (HPC) cluster (Intel Xeon E5—2670 2.6 GHz, 16 GB RAM). The computational time for all genes is about 36 h CPU time with 500 nodes. Then, the p-value of the proposed method can be computed analytically which is independently performed in each GWAS summary dataset. The computational time for each GWAS dataset is about 1 h CPU time with 10 nodes.

## Supplementary Information

Supplementary Information.

## Data Availability

The data that support the findings of this study are publically available and the links to the data are provided in the article.
