# Supplementary material for: Gene-based association tests using GWAS summary statistics and incorporating eQTL
Source: Sci Rep. 2022 Mar 3;12:3553. doi: 10.1038/s41598-022-07465-0 (PMC8894384; doi:10.1038/s41598-022-07465-0)

**Supplementary Materials**

**Table S1.** Information of the 18 genes used to obtain the number of replications in the estimation of $\mathbf{\Omega}$ and to evaluate type I error rates of Overall.

| **gene** | **position** | **# SNPs** | **Average LD** |
| --- | --- | --- | --- |
| *AGTRAP* | chr1: 11736084 - 11754802 | 23 | 12.72 |
| *TP53* | chr17: 7661779 - 7687538 | 25 | 13.78 |
| *OR8D2* | chr11:124319262-124320197 | 27 | 65.44 |
| *FNBP4* | chr11:47716494-47767443 | 29 | 108.34 |
| *HLA-DOA* | chr6: 33004182 - 33009591 | 38 | 13.21 |
| *C3orf22* | chr3: 126526999 - 126558965 | 40 | 24.29 |
| *GOSR1* | chr17: 17:30477362-30527592 | 40 | 78.42 |
| *LRRFIP2* | chr3:37052626-37183689 | 56 | 92.67 |
| *MCU* | chr10:72692131-72887694 | 56 | 144.73 |
| *C11orf49* | chr11:46936689-47164385 | 79 | 126.56 |
| *HLA-DOB* | chr6: 32812763 - 32820466 | 85 | 19.34 |
| *AKR1E2* | chr10: 4786629 - 4848062 | 89 | 16.05 |
| *DOCK3* | chr3:50674927-51384198 | 102 | 170.85 |
| *CCDC7* | chr10:32446140-32882874 | 117 | 56.56 |
| *SYNE2* | chr14: 63761899 - 64226433 | 174 | 36.13 |
| *UGT1A10* | chr2: 233636454 - 233773305 | 189 | 38.69 |
| *MCPH1* | chr8: 6406596 - 6648508 | 262 | 20.86 |
| *CDH13* | chr16: 82626965 - 83800640 | 359 | 17.25 |

*Notes:* “# SNPs” indicates the number of SNPs in the corresponding gene. “Average LD” indicates the average of LD scores of SNPs in the gene.

**Table S2.** Estimated type I error rates of Overall divided by the significance level for each of the 17 genes at significance levels of $1\times10^{-2}$, $1\times10^{-3}$, and $1\times10^{-4}$ with $2\times10^{5}$ replications.

| **Gene** | $1\times10^{-2}$ | $1\times10^{-3}$ | $1\times10^{-4}$ |
| --- | --- | --- | --- |
| *TP53* | 1.03 | 1.23 | 1.12 |
| *OR8D2* | 0.73 | 0.78 | 0.80 |
| *FNBP4* | 0.94 | 1.08 | 1.15 |
| *HLA-DOA* | 0.97 | 1.12 | 1.10 |
| *C3orf22* | 0.98 | 1.08 | 0.95 |
| *GOSR1* | 0.95 | 1.01 | 0.95 |
| *LRRFIP2* | 0.98 | 1.04 | 1.00 |
| *MCU* | 0.81 | 0.85 | 0.80 |
| *C11orf49* | 0.96 | 0.93 | 1.05 |
| *HLA-DOB* | 0.91 | 1.07 | 1.19 |
| *AKR1E2* | 1.10 | 1.07 | 1.21 |
| *DOCK3* | 0.98 | 0.94 | 0.65 |
| *CCDC7* | 1.01 | 1.20 | 1.10 |
| *SYNE2* | 0.94 | 1.03 | 1.00 |
| *UGT1A10* | 0.97 | 1.03 | 0.86 |
| *MCPH1* | 0.99 | 1.17 | 1.14 |
| *CDH13* | 1.10 | 1.13 | 1.12 |

**Figure S1.** The p-values to test if the estimated correlation matrix of p-values based on $B=10^{4}$ and the estimated correlation matrix of p-values based on $B_{0}$ are the same for the 18 genes. The red dotted line indicates the significant level 0.05.

**
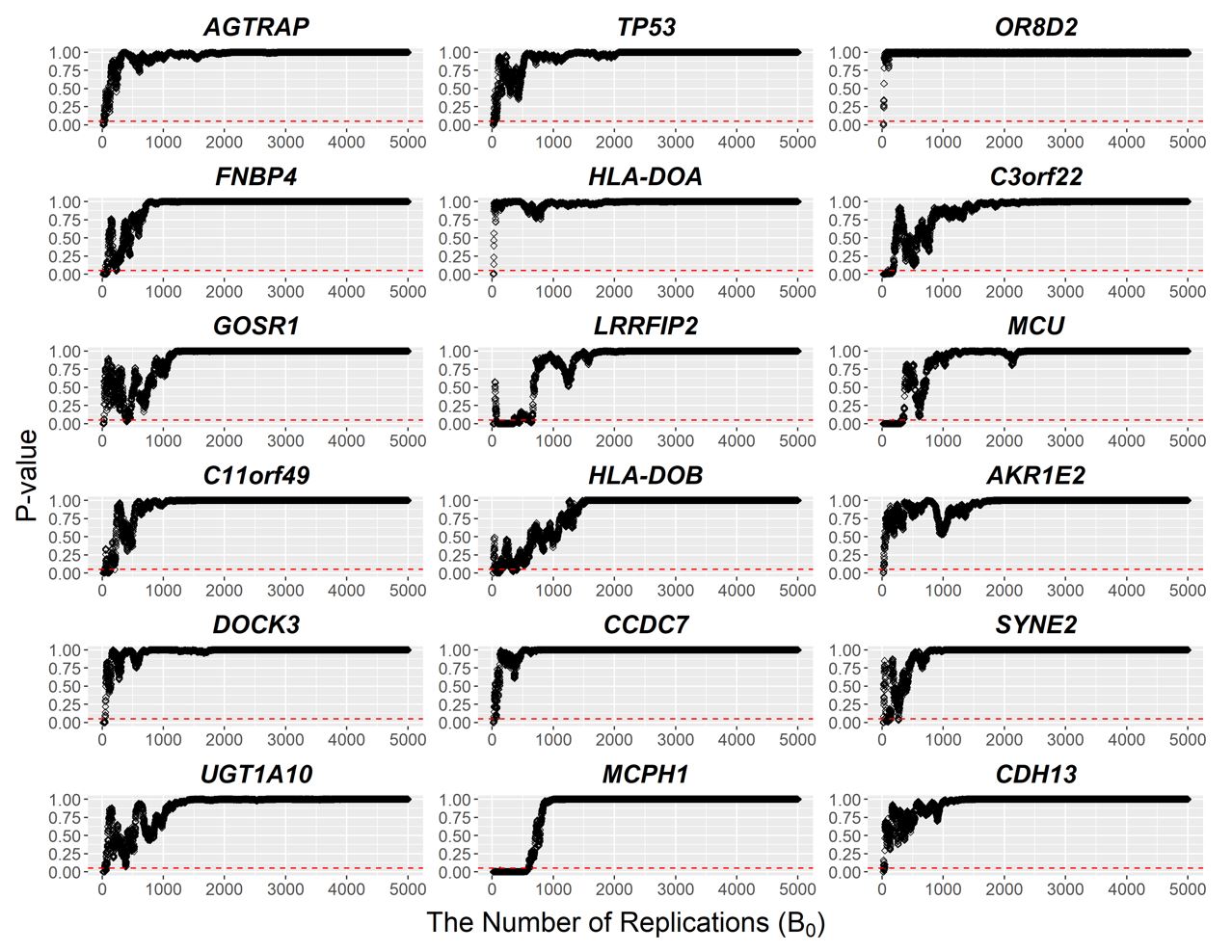
**

**Figure S2.** The LD block structures of gene *AGTRAP* (left) and gene *C3orf22* (right)*.*


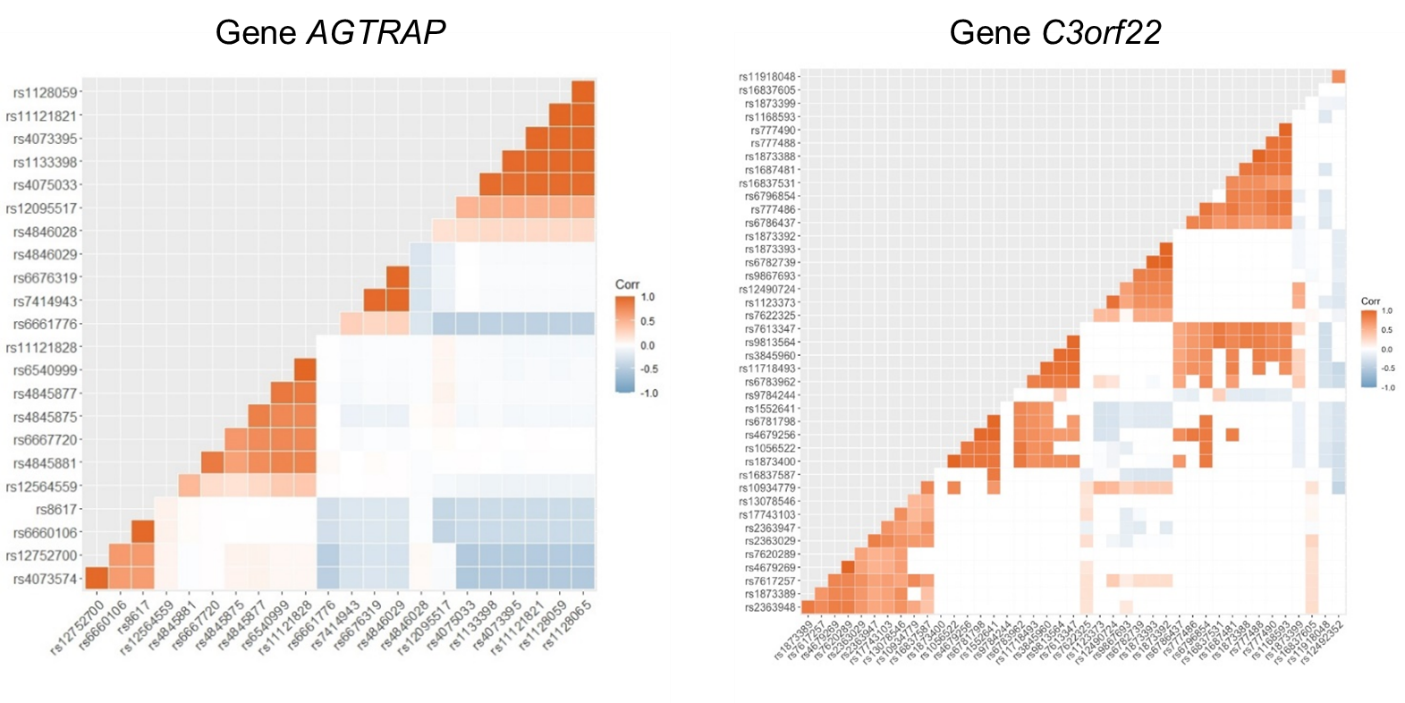


**Figure S3.** Estimated correlation matrix of p-values $\hat{\mathbf{\Omega}}$ for gene *AGTRAP*.


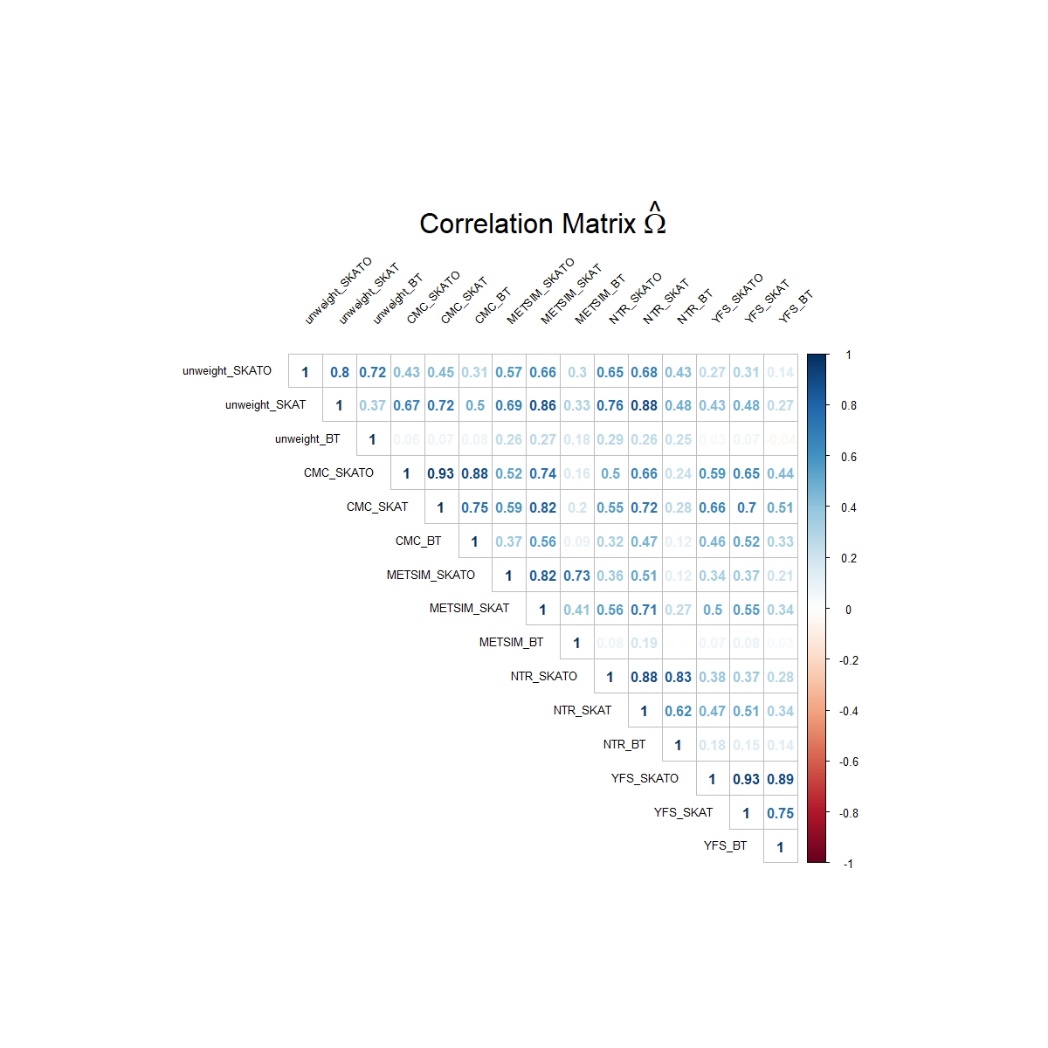


**Figure S4.** Power comparisons of gene-based association tests at $1.75\times10^{-6}$ significance level under Uni-directional effects ($\beta_{1}=\beta_{2}=\beta_{3}=\beta_{4}$) with $p_{causal}=\left(0.1,0.2,0.3,0.4\right)$ based on gene *C3orf22*. (a) Estimated power against phenotypic heritability $h_{p}^{2}$ with fixed expression heritability $h_{e}^{2}=0.2$; (b) Estimated power against expression heritability $h_{e}^{2}$ with fixed phenotypic heritability $h_{p}^{2}=0.2$.

*Scenario 1*: Uni-directional effects

(a) $h_{e}^{2}=0.2$ and $h_{p}^{2}=0.2,0.4,0.6,0.8$ (b) $h_{p}^{2}=0.2$ and $h_{e}^{2}=0.2,0.4,0.6,0.8$


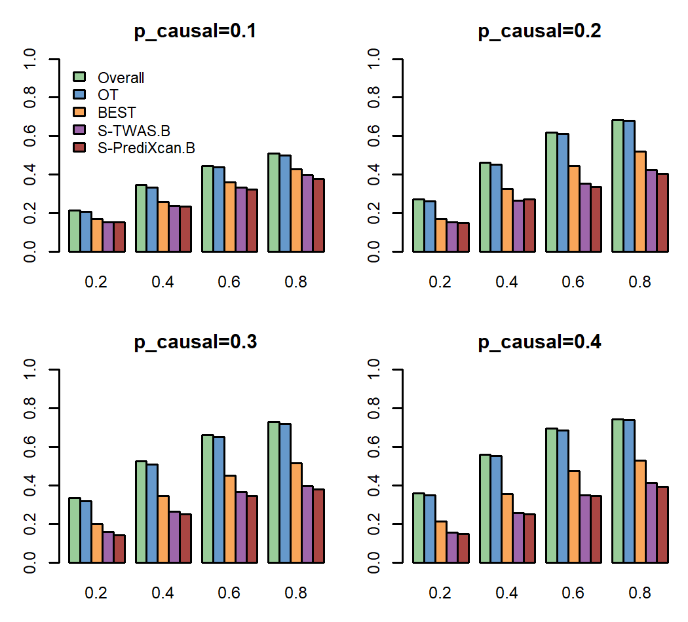

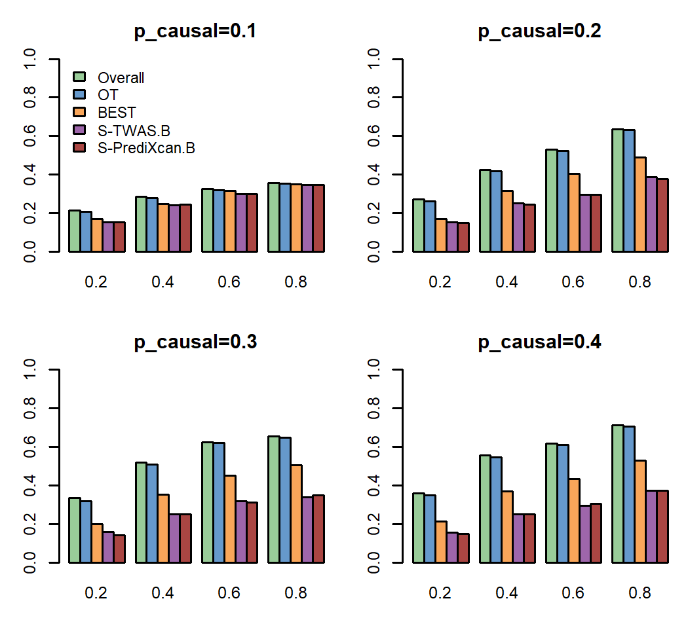


**Figure S5.** Power comparisons of gene-based association tests at $1.75\times10^{-6}$ significance level under Bi-directional effects ($\beta_{1}=\beta_{2}=-\beta_{3}=-\beta_{4}$) with $p_{causal}=\left(0.1,0.2,0.3,0.4\right)$ based on gene *C3orf22*. (a) Estimated power against phenotypic heritability $h_{p}^{2}$ with expression heritability $h_{e}^{2}=0.2$; (b) Estimated power against expression heritability $h_{e}^{2}$ with phenotypic heritability $h_{p}^{2}=0.2$.

*Scenario 2*: Bi-directional effects

(a) $h_{e}^{2}=0.2$ and $h_{p}^{2}=0.2,0.4,0.6,0.8$ (b) $h_{p}^{2}=0.2$ and $h_{e}^{2}=0.2,0.4,0.6,0.8$


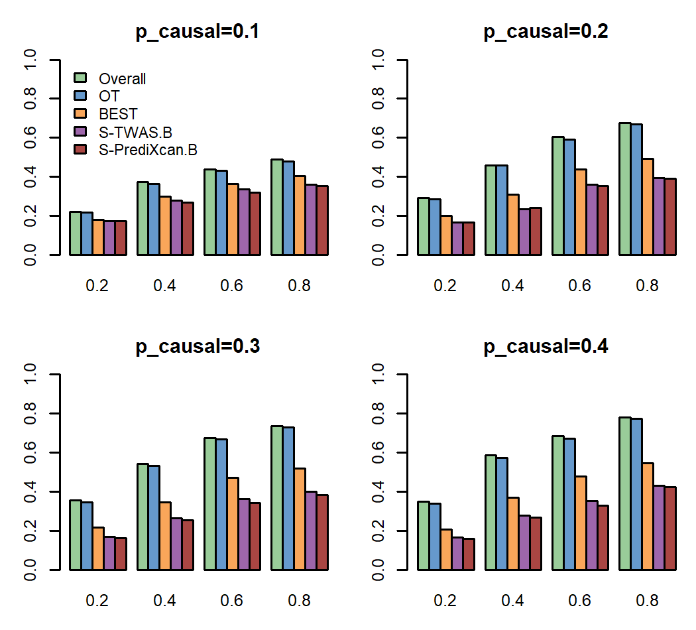

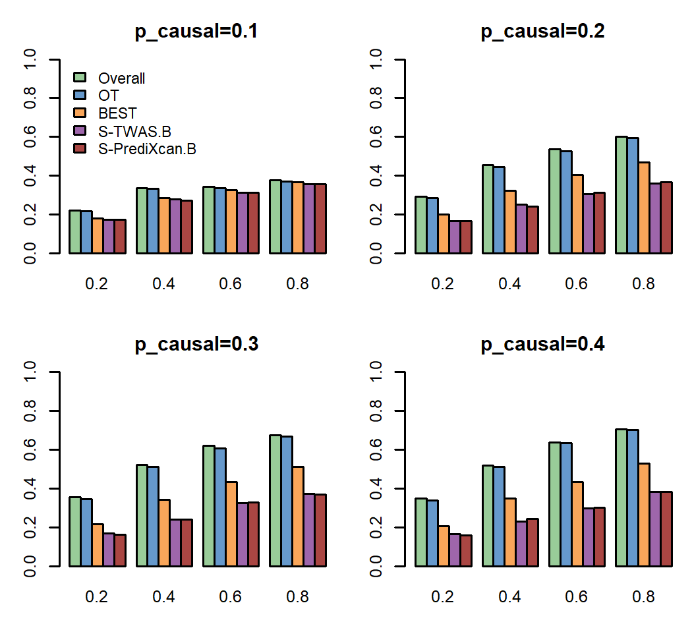


**Figure S6.** Power comparisons of gene-based association tests at $1.75\times10^{-6}$ significance level with $p_{causal}=\left(0.2,0.3\right)$ based on gene *C3orf22* with eQTL – derived weights from $K=20$ studies. Estimated power against phenotypic heritability $h_{p}^{2}$ with expression heritability $h_{e}^{2}=0.2$. (a) Uni-directional effects ($\beta_{1}=\cdots=\beta_{K}$); (b) Bi-directional effects ($\beta_{1}=\cdots=\beta_{K/2}=-\beta_{K/2+1}=\cdots=-\beta_{K}$).

$h_{e}^{2}=0.2$ and $h_{p}^{2}=0.2,0.4,0.6,0.8$

(a) Uni-directional effects (b) Bi-directional effects


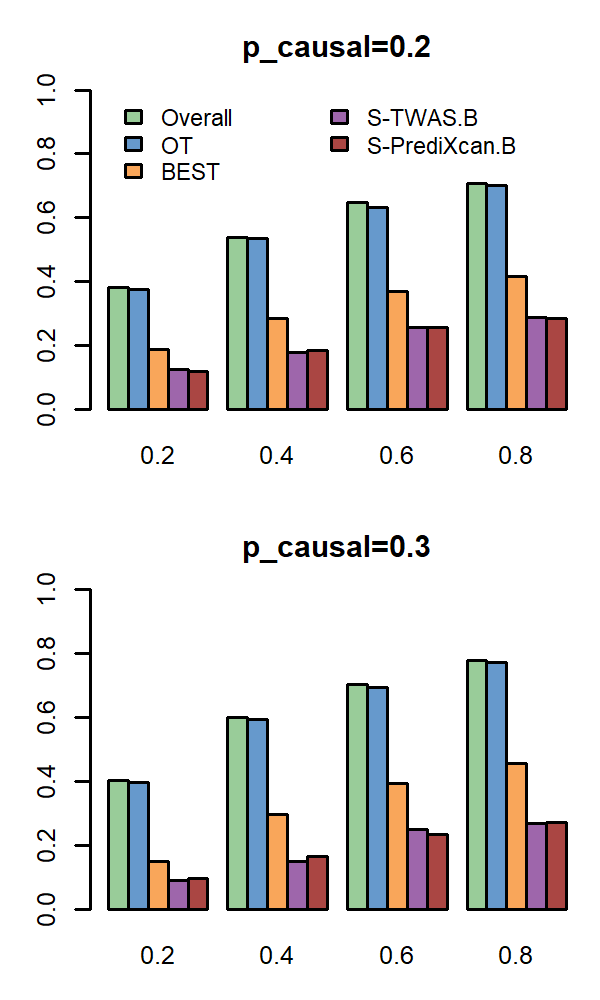

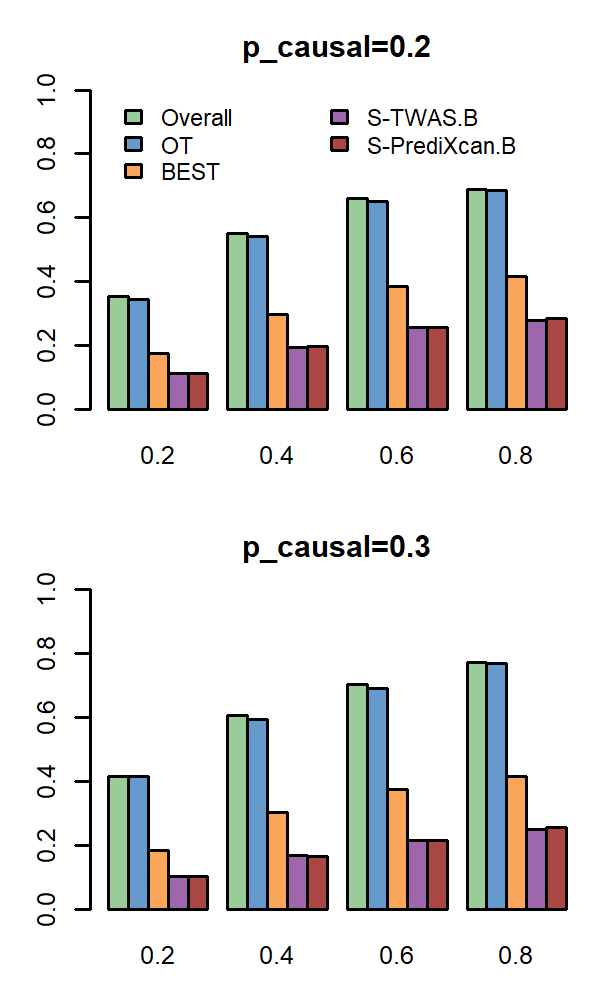


**Figure S7.** Power comparisons of gene-based association tests at $1.75\times10^{-6}$ significance level under Uni-directional effects with noise to the eQTL for $p_{causal}=\left(0.1,0.2,0.3,0.4\right)$ based on gene *C3orf22*. (a) Estimated power against phenotypic heritability $h_{p}^{2}$ with fixed expression heritability $h_{e}^{2}=0.2$; (b) Estimated power against expression heritability $h_{e}^{2}$ with fixed phenotypic heritability $h_{p}^{2}=0.2$.

*Scenario 1*: Uni-directional effects with noise to eQTL

(a) $h_{e}^{2}=0.2$ and $h_{p}^{2}=0.2,0.4,0.6,0.8$ (b) $h_{p}^{2}=0.2$ and $h_{e}^{2}=0.2,0.4,0.6,0.8$


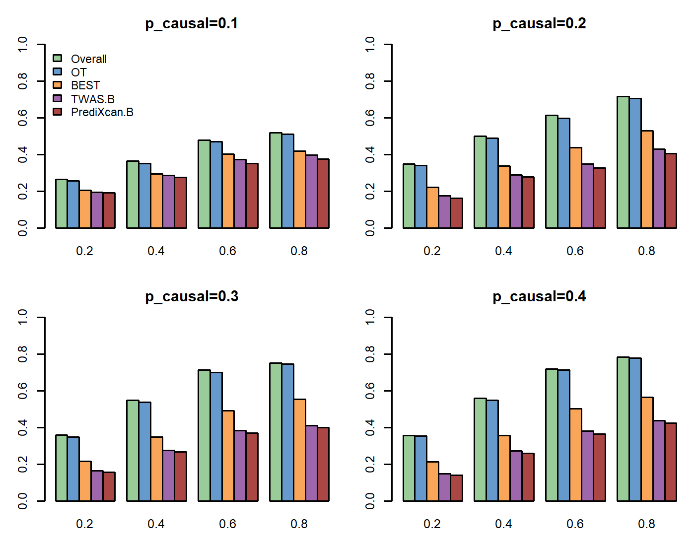

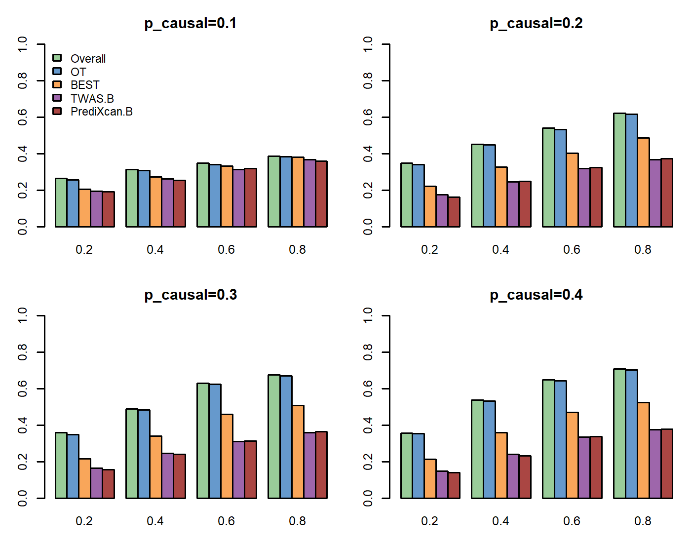


**Figure S8.** Power comparisons of gene-based association tests at $1.75\times10^{-6}$ significance level under Bi-directional effects with noise to the eQTL for $p_{causal}=\left(0.1,0.2,0.3,0.4\right)$ based on gene *C3orf22*. (a) Estimated power against phenotypic heritability $h_{p}^{2}$ with expression heritability $h_{e}^{2}=0.2$; (b) Estimated power against expression heritability $h_{e}^{2}$ with phenotypic heritability $h_{p}^{2}=0.2$.

*Scenario 2*: Bi-directional effects with noise to eQTL

(a) $h_{e}^{2}=0.2$ and $h_{p}^{2}=0.2,0.4,0.6,0.8$ (b) $h_{p}^{2}=0.2$ and $h_{e}^{2}=0.2,0.4,0.6,0.8$


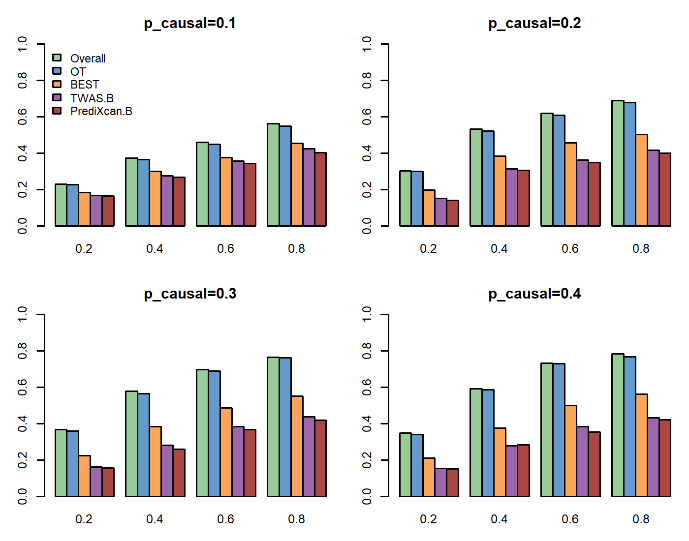

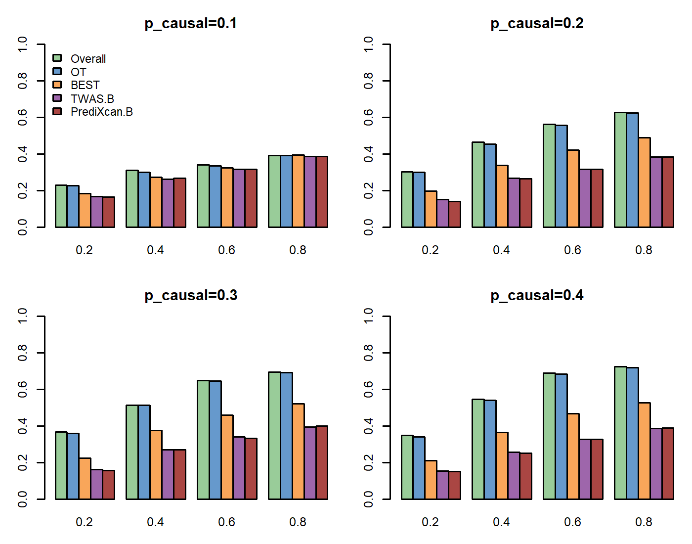


**Figure S9.** Estimated power against phenotypic heritability $h_{p}^{2}$ with expression heritability $h_{e}^{2}=0.05$ at $1.75\times10^{-6}$ significance level on gene C3orf22 with $p_{causal}=\left(0.1,0.2\right)$ and sample size of 100,000. (a) Uni-directional effects with noise to eQTL; (b) Bi-directional effects with noise to eQTL.

$h_{e}^{2}=0.05$ and $h_{p}^{2}=0.05,0.1,0.15,0.2$

(a) Uni-directional effects (b) Bi-directional effects


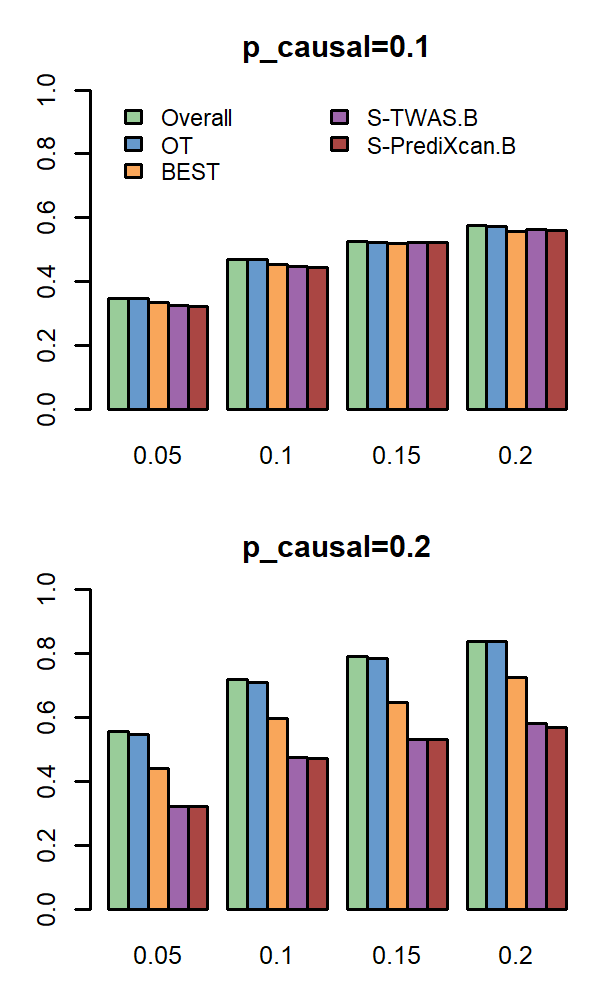

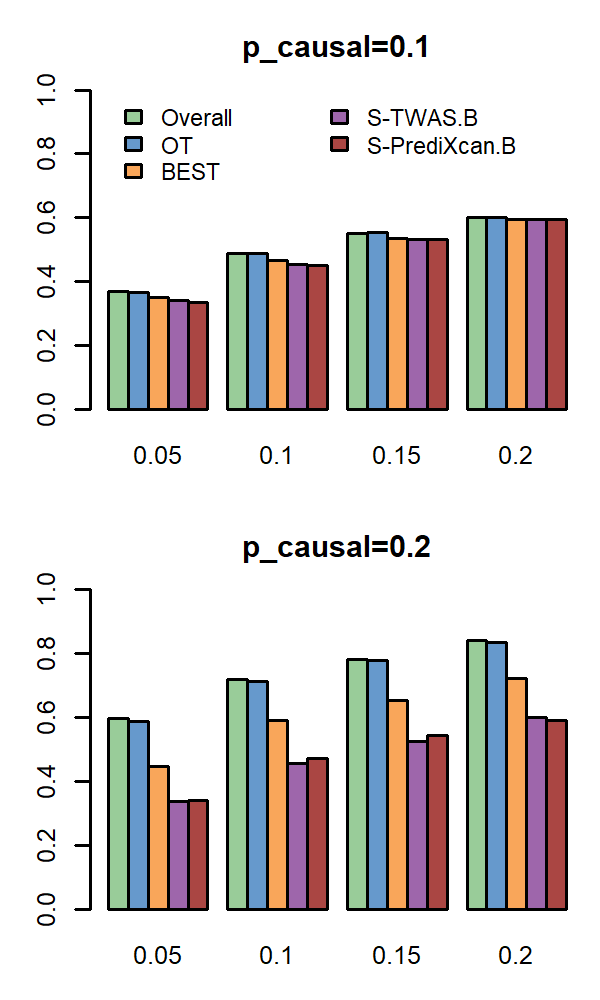


**Figure S10.** Power comparisons of Overall, OT, and BEST based on corrected and uncorrected LD structure on gene C3orf22 with $p_{causal}=\left(0.2,0.3\right)$ at $1.75\times10^{-6}$ significance level. (a) and (b): Uni-directional effects with noise to eQTL; (b) and (d): Bi-directional effects with noise to eQTL.

Uni-directional effects Bi-directional effects

(a) $h_{e}^{2}=0.2$ (b) $h_{p}^{2}=0.2$ (c) $h_{e}^{2}=0.2$ (d) $h_{p}^{2}=0.2$


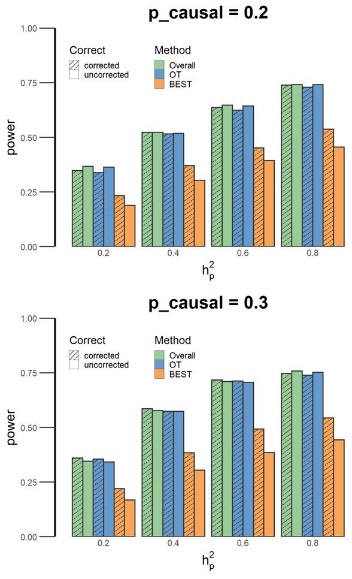

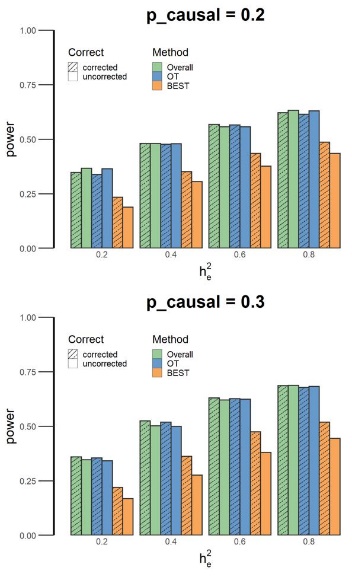

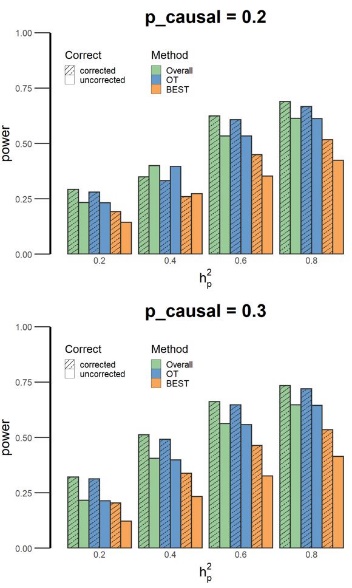

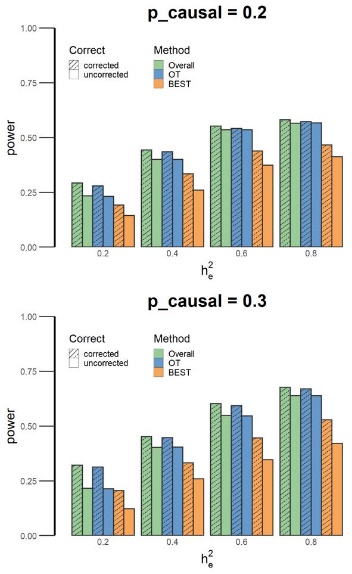

Supplement: Supplementary file 1 — Supplementary Information. [file 41598_2022_7465_MOESM1_ESM.docx]
